# Augmented region of interest for untargeted metabolomics mass spectrometry (AriumMS) of multi-platform-based CE-MS and LC-MS data

**DOI:** 10.1007/s00216-023-04715-6

**Published:** 2023-05-25

**Authors:** Lukas Naumann, Adrian Haun, Alisa Höchsmann, Michael Mohr, Martin Novák, Dirk Flottmann, Christian Neusüß

**Affiliations:** Department of Chemistry, Aalen University, Beethovenstraße 1, 73430 Aalen, Germany

**Keywords:** Augmented data evaluation, Mid-level data fusion, Multi-platform metabolomics, nanoCEasy, Capillary electrophoresis, Hydrophilic interaction liquid chromatography

## Abstract

In mass spectrometry (MS)-based metabolomics, there is a great need to combine different analytical separation techniques to cover metabolites of different polarities and apply appropriate multi-platform data processing. Here, we introduce AriumMS (augmented region of interest for untargeted metabolomics mass spectrometry) as a reliable toolbox for multi-platform metabolomics. AriumMS offers augmented data analysis of several separation techniques utilizing a region-of-interest algorithm. To demonstrate the capabilities of AriumMS, five datasets were combined. This includes three newly developed capillary electrophoresis (CE)-Orbitrap MS methods using the recently introduced nanoCEasy CE-MS interface and two hydrophilic interaction liquid chromatography (HILIC)-Orbitrap MS methods. AriumMS provides a novel mid-level data fusion approach for multi-platform data analysis to simplify and speed up multi-platform data processing and evaluation. The key feature of AriumMS lies in the optimized data processing strategy, including parallel processing of datasets and flexible parameterization for processing of individual separation methods with different peak characteristics. As a case study, *Saccharomyces cerevisiae* (yeast) was treated with a growth inhibitor, and AriumMS successfully differentiated the metabolome based on the augmented multi-platform CE-MS and HILIC-MS investigation. As a result, AriumMS is proposed as a powerful tool to improve the accuracy and selectivity of metabolome analysis through the integration of several HILIC-MS/CE-MS techniques.

**Graphical Abstract:**

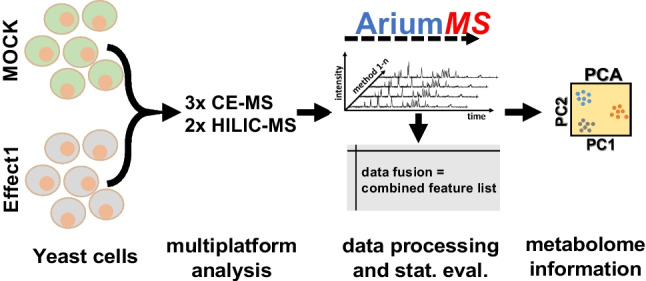

**Supplementary Information:**

The online version contains supplementary material available at 10.1007/s00216-023-04715-6.

## Introduction

Owing to the inherent chemical diversity and the large size of the metabolome, there is no universal technique that can be used to assess the entire metabolome, i.e., “one size does not fit all” [1, 2]. Nevertheless, multi-platform metabolomics workflows based on mass spectrometry (MS) are able to enhance metabolome coverage.

Typically, scientists employ high-resolution electrospray ionization–MS (ESI-MS) with the possibility of MS/MS experiments such as quadrupole time-of-flight (QTOF) or Orbitrap MS [3–5]. Depending on the type of metabolites to be measured (polar vs. nonpolar) and limitations concerning time and sample amount, different separation techniques can be applied for the analysis to expand the metabolome coverage [1]. These are reversed-phase liquid chromatography (RP-LC) [3], hydrophilic interaction liquid chromatography (HILIC) [6], capillary electrophoresis (CE) [7], and gas chromatography (GC) [8] coupled to high-resolution MS [3, 9]. The analytical gold standard in proteomics and metabolomics is RP-LC-MS because of its extended dynamic concentration range, sensitivity, retention time reproducibility, and ease of use [6]. Since RP-LC does not retain very well a wide variety of highly polar and ionizable metabolites, HILIC is a valuable alternative [6]. HILIC is driven by molecular interactions and the partition of analytes between the hydrophobic mobile phase and the hydrophilic stationary phase [10]. Significant technological advances in HILIC over the last two decades, such as the commercialization of dedicated HILIC columns, have aided the implementation of HILIC in proteomics and metabolomics [6]. Overall, this has resulted in significant analytical improvements (e.g., sensitivity, analyte coverage, throughput, analysis speed, and resolution), and thus HILIC offers excellent opportunities for the analysis of polar and/or ionizable metabolites [6].

Since many metabolites, especially those of central carbon metabolism, contain charged amino, hydroxyl, carboxyl, and phosphate groups, they are especially suitable for CE-MS analysis [10]. Electrophoretic-driven separation approaches offer several advantages for the separation of charged compounds, like efficient separation, high resolving power, low solvent, and sample consumption. Since CE separation is based on differences in ion mobilities [10, 11], different compositions of background electrolytes (BGE), especially regarding pH, lead to different selectivities. In this way, CE-MS analysis has been frequently applied using acidic BGEs for cation analysis [12–14] and basic BGEs for anion analysis [15–17]. In order to improve the sensitivity for metabolite analysis by CE-MS, nanoESI interfaces have recently been used, including the porous tip interface [18]. Nanosheath–liquid interfaces are of high interest as well, due to additional flexibility and robustness [19]. Most recently, we introduced the nanoCEasy interface adding ease-of-use and the capability of valve functionality by the two-capillary approach (e.g., for capillary reconditioning between runs) [20–22].

Metabolomics data evaluation is usually based on two major approaches: target and non-target data evaluation [3]. Target-based data evaluation is hypothesis-driven and focuses with high analytical sensitivity on standard mixtures for their assignment and interpretation, such as concentration and appearance [3, 23]. Non-target metabolomics is an exploratory, hypothesis-generating data evaluation workflow [3]. This approach is a common choice as a first step within a data evaluation, to capture and monitor a broad range of molecular content and retrieve as much chemical information as possible without any prior knowledge [3]. Examples of multi-platform metabolomics can be found in previous publications [7, 24–28]. Most of them use a target/non-target approach based on different data processing workflows for each analytical platform.

Since LC-MS and CE-MS offer comprehensive information of the metabolome, a combined multi-platform non-targeted data evaluation based on a data fusion approach offers a single chemometric result for enhanced statistical prediction and metabolic coverage [29–31]. The fusion of separation methods coupled with MS detection is challenging due to the multivariate nature of the data (i.e., a very high variables-to-sample ratio, and shift in migration times during sequences) [31, 32]. Hence, an augmented data evaluation of comprehensive analytical workflows enhances the feature capacity by combining different selectivities, thereby allowing a better characterization of phenotypes.

Data augmentation describes the combination of several datasets into one. Three cases can be distinguished [31, 33, 34]: Low-level fusion is applied before any data reduction, and mid-level fusion after feature extraction, whereas high-level data fusion combines models after data analysis [35]. Mid-level data fusion is based on removing irrelevant information, such as artifacts and noise, from each dataset. The resulting dimensionality reduction decreases computation time and can produce more robust models [36].

Region-of-interest (ROI) analysis is the approach of choice and significantly reduces both the amount of data—without loss of relevant information—and processing time. Only data points that have a minimum intensity and a minimum abundance within the measurement are included in an ROI [37]. Peaks are then detected, integrated, and labeled (m/z, retention time, peak area, and height) in the obtained ROIs. Various filters (e.g., contaminant filter, isotope, and adduct filter) are then used to remove false-positive features from the feature list. This method is widely used in the web-based tools XCMS Online [38] and MetaboAnalyst [39] as well as in various software packages such as MetaboAnalystR [40] or the open-source MZmine [41]. However, the focus of these programs is not on the augmentation of different separation techniques. For example, it is not possible to select different preprocessing settings for different data, which is essential for different separation systems. With XCMS Online and MZmine, files of different origins must be processed separately and augmented manually afterward.

Here, we present the novel open-source AriumMS (augmented region of interest for untargeted metabolomics mass spectrometry) software to challenge the multi-platform metabolomics data analysis in combination with new methods for the analysis of polar metabolites by different CE and HILIC separation techniques. AriumMS contains a universal and user-friendly toolbox, capable of handling multi-platform datasets. AriumMS offers automated batch processing with flexible processing options and a graphical user interface. The suitability of this tool for multi-platform metabolomics is demonstrated within a comparative study of metabolic standard mixtures and different yeast phenotypes. Therefore, the metabolic standard mixtures and the yeast extracts were measured within a multi-platform approach combining HILIC-MS (ESI positive/ESI negative) with three CE-MS methods applying our recently introduced nanoCEasy interface. A cationic CE-MS method was complemented by two CE-MS methods to cover a wide range of anionic metabolites.

## Materials and methods

### Materials

The amino acid standard (1 nmol/µL in 0.1 M hydrochloric acid) was obtained from Agilent Technologies (Santa Clara, CA, USA). The internal standards and metabolites used were obtained from Sigma-Aldrich (St. Louis, MO, USA). Sugars (nucleotide sugars, phosphate sugars) were purchased from Biosynth Carbosynth (Staad, Switzerland). Synthetic Dextrose Minimal Medium (SD, synthetic minimal medium) was obtained from Carl Roth (Karlsruhe, Germany). Standard materials and composition of the metabolomics standard can be found in the supplements.

### Yeast growth and sample preparation

Production of yeast liquid cultures was carried out with *Saccharomyces cerevisiae* strain CEN.PK122 [42], starting from a single colony grown on SD plates. Growth took place in an incubation shaker in a 5 L baffle flask under controlled conditions (30 °C, 123 rpm, 16 h). SD medium was used as a basal medium. The culture was split into two cultures (Mock and Effect1) at 0.5 optical density. Cell line Effect1 (160 mL) contained 160 µL 35 mM halogenated indole dilution in dimethyl sulfoxide (DMSO) to induce the effect. In order to determine the induced effect of the halogenated indole exactly, Mock (160 mL) as a reference was treated exactly the same as Effect1 (160 µL DMSO), without adding the halogenated indole. Incubation at 30 °C and 170 rpm monitored by optical density readings every 30–60 min was performed until the inhibition of the cell growth became apparent. Thereafter, cells were harvested and centrifuged. The cell pellets were washed and shock-frozen (at −80 °C). Further sample preparation is given in the supplements.

### Capillary electrophoresis

CE-ESI-MS was performed with a 7100 capillary electrophoresis system (model no. G7100A) from Agilent Technologies (Waldbronn, Germany) coupled with an Orbitrap Fusion Lumos mass spectrometer (Thermo Fisher Scientific, San Jose CA, USA) using the nanoCEasy interface [20]. Bare fused silica capillaries with 50/100 µm inner diameter and 360/240 µm outer diameter (separation/sheath liquid capillary) were obtained from Polymicro Technologies (Phoenix, AZ, USA). Separation capillaries had a length of 90 cm and were etched with hydrofluoric acid to about an 80–100 µm outer diameter. Three CE-MS methods have been used with the following background electrolyte (BGE) and sheath liquid (SL) compositions: anionic (acidic): 0.2 M formic acid pH 2.1 (BGE) and 50:50 (v/v) 2-propanol/water with 0.5% (v/v) of formic acid (SL); anionic (alkaline): 30 mM ammonium acetate pH 8.5 (BGE) and 50:50 (v/v) 2-propanol/water with 2.5 mM ammonium acetate (SL); and cationic (acidic): 1 M formic acid containing 10% 2-propanol pH 1.7 (BGE) and 50:50 (v/v) 2-propanol/water with 0.5% (v/v) of formic acid (SL). For each measurement, the capillary was preconditioned by flushing with BGE for 5 min. For the alkaline CE method, the capillary was additionally primed for 5 min applying 30 kV, and again flushing with BGE for 5 min. Samples were injected hydrodynamically with 40 mbar for 27 s (1% capillary volume). Separation was performed by applying a potential of +30 kV (cationic acidic and anionic alkaline BGE method) or −30 kV (anionic acidic BGE method) to the capillary inlet. SL was delivered via a syringe pump (100 series, kdScientific, Hilliston, MA, USA) with a flow rate of 10 µL/min, equipped with a 5 mL syringe (SGE Analytical Science, Melbourne, Australia). The anionic acidic and anionic alkaline CE-MS methods were detected in ESI negative mode. The cationic acidic CE-MS method was detected in ESI positive mode. Source parameters for Orbitrap were set to −1700 V/−2000 V/1900 V (anionic acidic/anionic alkaline/cationic acidic) spray voltage, 3 a.u. (arbitrary units) sheath gas, 0 a.u. aux gas, and 300 °C ion transfer tube.

### Hydrophilic interaction liquid chromatography

A Dionex UltiMate 3000 (Dionex, Sunnyvale, CA, USA) high-performance liquid chromatography (HPLC) system equipped with a VDSpher PUR 100 HILIC guard and separation column (4.2 × 10 mm and 150 × 3 mm, 5 µm particle size, VDS optilab Chromatographietechnik GmbH, Berlin, Germany) heated to 30 °C was used. Mobile phase A was composed of H_2_O, acetonitrile (95/5 v/v), and 5 mM ammonium acetate, and mobile phase B was composed of H_2_O, acetonitrile (5/95 v/v), and 5 mM ammonium acetate. The sample injection volume was 3 µL, and the run time was 35 min. The gradient started at 10% A, followed by a 15-min linear gradient from 10 to 60% A, and hold for 5 min. Column re-equilibration was performed for 15 min at 10% A. The flow rate was 300 µL/min. The LC was coupled to the Orbitrap with the respective standard heated electrospray ionization (HESI) source and sprayer. The Orbitrap source parameters were set to 3500 V positive/negative spray voltage, 50 a.u. sheath gas, 10 a.u. aux gas, 325 °C transfer tube, and 350 °C vaporizer temperature.

### Mass spectrometry

For mass spectrometry, an Orbitrap Fusion Lumos mass spectrometer (Thermo Fisher Scientific, San Jose CA, USA) was used in either positive or negative ion mode, with a scan range of 100–700 m/z. Resolving power was set to 60,000, accumulation time to 50 ms, automatic gain control (AGC) target to “standard,” 35% RF lens, and 1 micro scan. Data-dependent MS/MS experiments with 0.6 s cycle time were performed. Filters were an intensity threshold at 2E4, exclusion after a single occurrence for 10 s, and isotope exclusion. Data-dependent MS/MS Orbitrap higher-energy collisional dissociation fragmentation (HCD) parameters were isolation width of 1.5 da, 20/35/50% HCD power, Orbitrap resolution of 30,000, 54 ms accumulation time, AGC target set to “standard,” 35% RF lens, and 1 micro scan.

### Data evaluation and interpretation

Data acquisition was performed using a Thermo Scientific Xcalibur 4.1.50 and Orbitrap Tribrid MS Series Instrument Control Software version 3.2 (Thermo Fisher Scientific, San Jose CA, USA). Extraction of ion traces for the evaluation of separation methods was done with FreeStyle 1.5.93.34 (Thermo Fisher Scientific, San Jose CA, USA). MSconvert 3 (ProteoWizard, Palo Alto CA, USA) [43] was used for the initial data conversion. Non-target data evaluation was performed with AriumMS 1.0.0 (https://github.com/AdrianHaun/AriumMS/). Software and parameters for evaluation are given in the Supporting Information (supplement Table S1 and Table S2).

## Results and discussion

### Study design

**Fig. 1:**
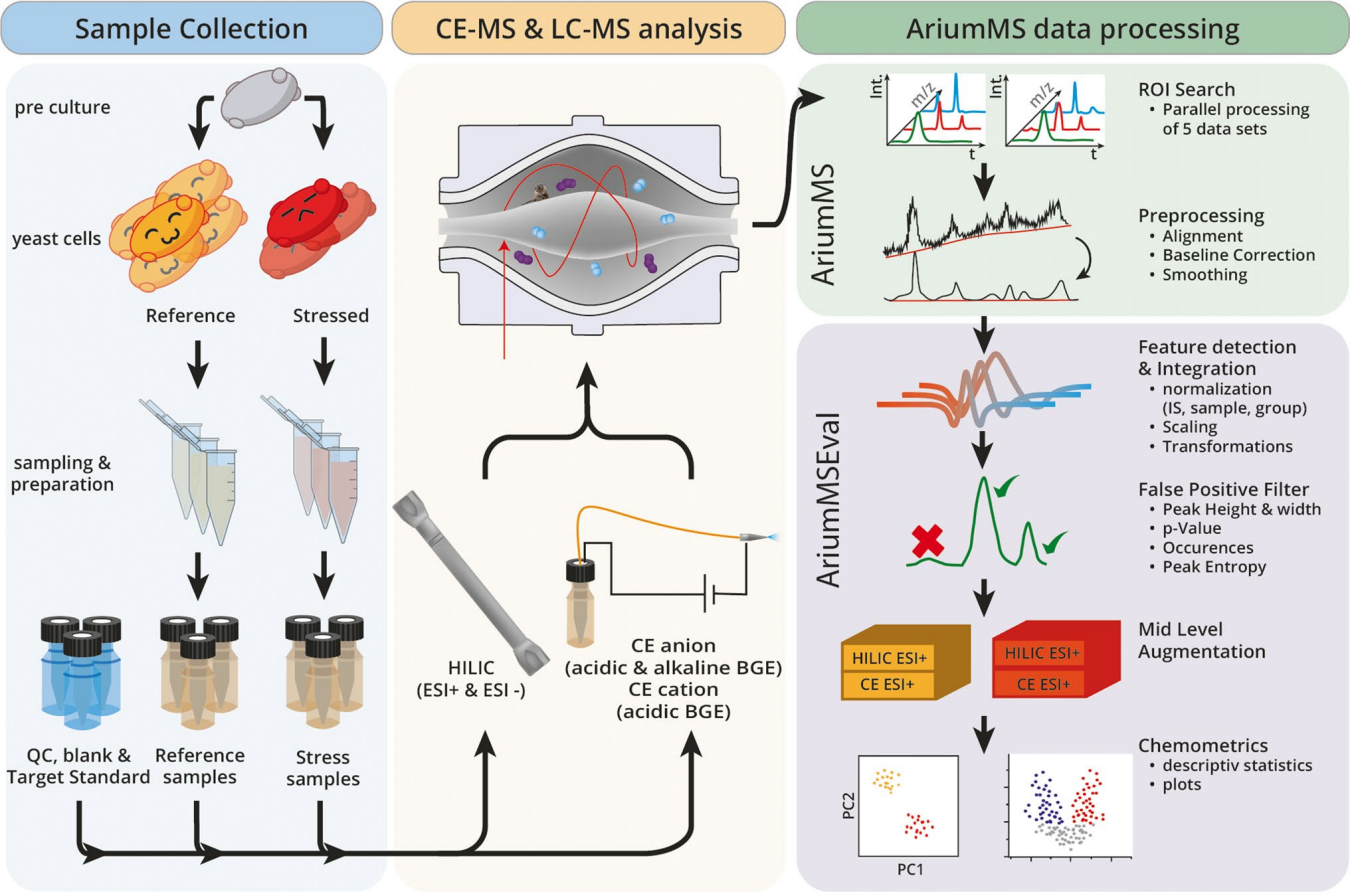
Multi-platform metabolomics workflow overview, containing all steps of sampling, analysis, and AriumMS workflow

In order to present AriumMS as a toolbox for the challenge of multi-platform metabolomics data analysis, metabolite standards and yeast extract samples were measured with five analytical methods. The metabolite standard that was used contained 36 metabolites, covering important polar/ionic substance classes (mass range of 100–665 Da). The yeast extracts contained the metabolic information of the induced effect by a halogenated indole treatment. To analyze polar and/or ionic metabolites of interest within the samples, two HILIC-MS and three CE-MS methods have been developed. In order to determine optimal AriumMS data processing parameters for the generated datasets of the five analytical methods, a D-optimal design of experiment (DOE) was applied for software parameter screening and optimization. Furthermore, the feature generation of AriumMS was validated. This was followed by a multi-platform metabolomics data analysis of the yeast extracts. The complete analytical workflow is shown in Fig. 1.

### Evaluation of the analytical methods

**Fig. 2:**
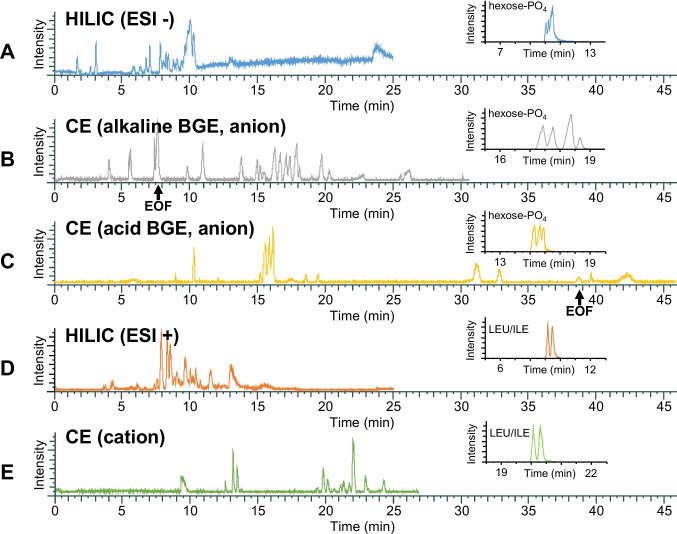
Comparison of different analytical methods using the measurements of the metabolomics standard that contains 40 substances. The BPC/Es are shown at **A**–**E** containing a zoom to the separation of either the four hexose phosphates (anion methods) or L-leucine and L-isoleucine (cation methods). (**A**) HILIC-MS anion, blue; (**B**) CE-MS anion alkaline, gray; (**C**) CE-MS anion acidic, yellow; (**D**) HILIC-MS cation, orange; (**E**) CE-MS cation, acidic

The standard contained a total of 36 typical polar metabolites and four internal standards, including yeast metabolites, amino acids, hexoses, hexose phosphates, and nucleotide sugars. Anionic, cationic, zwitterionic, and uncharged species were represented (Table 1). To determine the overall capabilities of the five different analytical methods regarding the number of detected analytes and duration of analysis, six repetitions of the metabolomics standard were measured with each method. The five separation methods were evaluated regarding the number of detectable analytes, their migration time (MT)/retention time (RT), and their separation efficiency (for further details, see supporting information, Fig. 2a–e, Table 1). These five separation methods offered overlapping and complementary information on the metabolite standard, as shown in Fig. 2 and Table 1: The two HILIC methods covered most of the metabolites, i.e., 25 of 36 in ESI+ and 27 of 36 in ESI−, respectively, and when they were combined, 30 of 36 metabolites of the standard were able to be detected. Some multi-carboxylic acids and basic amino acids were not detected, and isomeric hexose phosphates were not baseline-separated. The selectivity of the CE-MS methods used was higher. The anionic alkaline CE-MS method was able to detect 30 out of 36 metabolites over a period of 30 min. Seventeen metabolites (such as neutral amino acids) co-migrated with the electroosmotic flow (EOF, no separation) (see Table 1). The anionic acidic CE-MS method was capable of analyzing phosphates and dicarboxylic acids and covered 15 of 36 analytes (four co-migrating neutrals) over a period of 45 min (Table 1). Using the cation CE-MS method, 17 of 36 metabolites were able to be detected. Neutral metabolites, such as hexoses and caffeine, were not detected by any of the CE-MS methods. In summary, when the three CE-MS methods were combined, CE-MS was able to detect 32 of 36 metabolites. When all five analytical methods were applied, all metabolites of the standard were detectable (Table 1). Apart from the difference in selectivity, HILIC-MS and CE-MS each had distinct advantages: HILIC-MS exhibited a higher retention time reproducibility and a higher degree of automation, while CE-MS required a smaller sample volume and showed more efficient separation with sharper peaks.

**Table 1:** List of all target analytes of the metabolomics standard

Group	Analyte		Anion							Cation				
	ID	Name		HILIC		CE (alkaline BGE)		CE (acidic BGE)			HILIC		CE	
			[M−H]^−^	RT ± sd [min]	AriumMS	MT ± sd [min]	AriumMS	MT ± sd [min]	AriumMS	[M+H]^+^	RT ± sd [min]	AriumMS	MT ± sd [min]	AriumMS
Amino acid	1	L-serine	104.0348	10.8 ± 0.1	✓	7.7* ± 0.3	✓	-	-	106.0504	10.8 ± 0.0	✓	19.5 ± 0.5	✓
	2	L-proline	114.0555	11.6 ± 0.1	✓	7.8* ± 0.3	✓	-	-	116.0712	11.8 ± 0.1	✓	21.5 ± 0.6	✓
	3	L-valine	116.0712	10.1 ± 0.1	✓	7.8* ± 0.3	✓	-	-	118.0869	10.1 ± 0.0	✓	19.6 ± 0.5	✓
	4	L-threonine	118.0504	10.6 ± 0.1	✓	7.8* ± 0.3	✓	-	-	120.0661	10.6 ± 0.0	✓	20.8 ± 0.6	✓
	5	L-cysteine	120.0119	-	-	7.8* ± 0.3	✓	-	-	122.0276	13.2 ± 0.1	✓	22.3 ± 0.7	✗
	6	L-leucine	130.0869	9.1 ± 0.0	✓	7.8* ± 0.3	✓	-	-	132.1025	9.1 ± 0.0	✓	20.1 ± 0.6	✓
	7	L-isoleucine	130.0869	9.4 ± 0.0	✓	7.8* ± 0.3	✓	-	-	132.1025	9.4 ± 0.0	✓	20.4 ± 0.6	✓
	8	L-aspartic acid	132.0297	9.9 ± 0.1	✓	15.9 ± 1.1	✓	38.6 ± 1.6	✗	134.0454	10.0 ± 0.0	✓	23.4 ± 0.7	✓
	9	L-lysine	145.0978	-	-	5.4 ± 0.1	✓	-	-	147.1134	15.5 ± 0.5	✗	12.7 ± 0.2	✓
	10	L-glutamic acid	146.0454	10.2 ± 0.1	✓	14.5 ± 0.9	✓	-	-	148.0610	10.2 ± 0.0	✓	22.0 ± 0.7	✓
	11	L-methionine	148.0433	9.3 ± 0.1	✓	7.8* ± 0.3	✓	-	-	150.0589	9.3 ± 0.0	✓	21.3 ± 0.6	✓
	12	L-histidine	154.0617	-	-	7.4 ± 0.2	✓	-	-	156.0773	-	-	13.6 ± 0.3	✓
	13	L-phenylalanine	164.0712	8.7 ± 0.4	✓	7.8* ± 0.3	✓	-	-	166.0869	8.6 ± 0.0	✓	22.2 ± 0.7	✓
	14	L-arginine	173.1039	-	-	5.5 ± 0.1	✓	-	-	175.1196	-	-	13.3 ± 0.3	✓
	15	L-tyrosine	180.0661	8.8 ± 0.0	✓	7.8* ± 0.3	✓	-	-	182.0818	8.8 ± 0.0	✓	23.2 ± 0.7	✓
	16	L-tryptophan	203.0821	8.1 ± 0.0	✓	7.8* ± 0.3	✓	-	-	205.0978	8.1 ± 0.0	✓	22.3 ± 0.7	✓
Internal std	17	benzenesulfinic acid	141.0010	3.5 ± 0.1	✗	-	-	12.8 ± 0.2	✗	143.0167	-	-	-	-
	18	2-nitrobenzoic acid	166.0140	3.1 ± 0.0	✓	16.3 ± 1.3	✓	19.2 ± 0.4	✓	168.0297	-	-	-	-
	19	methionine sulfone	180.0331	9.8 ± 0.1	✓	7.7* ± 0.3	✓	-		182.0487	9.9 ± 0.0	✓	24.6 ± 0.8	✓
	20	pentetic acid	392.1306	10.3 ± 0.1	✓	30.7 ± 5.8	✗	-		394.1462	10.4 ± 0.0	✓	-	-
Metabolites	21	succinate	117.0188	7.7 ± 1.0	✓	-	-	37.7 ± 1.5	✓	119.0345	-	-	-	-
	22	nicotinic acid	122.0242	6.3 ± 0.1	✓	17.4 ± 1.4	✓	19.2 ± 0.4	✓	124.0399	6.4 ± 0.0	✓	20.0 ± 0.6	✓
	23	tartaric acid	149.0086	-	-	-	-	30.4 ± 1.0	✓	151.0243	-	-	-	-
	24	citrate; citric acid	191.0192	-	-	-	-	32.1 ± 1.1	✗	193.0348	-	-	-	-
	25	caffeine	193.0726	-	-	-	-	-	-	195.0882	4.0 ± 0.0	✓	-	-
	26	ATP	505.9879	10.2 ± 0.1	✗	25.3 ± 2.8	✗	-	-	508.0036	10.4 ± 0.0	✗	-	-
	27	NAD	662.1013	10.5 ± 0.0	✓	10.1 ± 0.5	✓	38.5 ± 1.6	✗	664.1170	10.6 ± 0.0	✓	-	-
	28	NADH	664.1170	8.6 ± 0.0	✓	13.6 ± 0.9	✗	-	-	666.1327	8.7 ± 0.0	✓	-	-
Carbohydrates	29	L-fuc., 6-deoxy-L-gal	163.0607	-	-	7.8* ± 0.3	✓	38.9^#^ ± 1.7	✗	165.0763	-	-	-	-
	30	D-mannose	179.0556	6.5 ± 0.2	✓	7.8* ± 0.3	✓	38.9^#^ ± 1.7	✗	181.0713	-	-	-	-
	31	α-D-glucose	179.0556	6.8 ± 0.2	✓	7.8* ± 0.3	✓	38.9^#^ ± 1.7	✗	181.0713	-	-	-	-
	32	α-D-galactose	179.0556	7.5 ± 0.6	✓	7.8* ± 0.3	✓	38.9^#^ ± 1.7	✗	181.0713	-	-	-	-
	33	D-mannose-1-PO_4_	259.0219	10.3 ± 0.0	✓	21.9 ± 1.3	✓	15.9 ± 0.2	✓	261.0376	10.3 ± 0.0	✗	-	-
	34	β-D-fructose-6-PO_4_	259.0219	10.1 ± 0.0	✓	19.4 ± 1.1	✓	15.8 ± 0.2	✓	261.0376	10.1 ± 0.0	✗	-	-
	35	α-D-glucose 1-PO_4_	259.0219	9.9 ± 0.0	✓	19.0 ± 1.1	✓	15.5 ± 0.2	✓	261.0376	9.9 ± 0.0	✗	-	-
	36	α-D-galactose-1-PO_4_	259.0219	10.3 ± 0.0	✓	18.1 ± 0.9	✓	15.5 ± 0.2	✓	261.0376	10.3 ± 0.0	✓	-	-
	37	N-acetylneuraminate	307.0909	-	-	7.7* ± 0.3	✓	-	-	309.1066	-	-	-	-
	38	UDP-glucose	565.0472	7.5 ± 0.1	✓	14.7 ± 0.9	✓	11.7 ± 0.8		567.0629	7.6 ± 0.0	✓	-	-
	39	GDP-L-fucose	588.0744	8.7 ± 0.3	✓	-	-	-	-	590.0901	9.0 ± 0.0	✓	-	-
	40	CMP-N-acetylneuraminate	613.1395	9.0 ± 0.0	✓	-	-	-	-	615.1552	-	-	-	-

If the peaks of the analytes fulfill the general criteria for peak detection (maximum peak width ≤ 2 min, peak intensity ≥ 5E4), then the retention time ± standard deviation (sd) is given. If an analyte is not detected by the method itself or does not fulfill the criteria for peak detection, it is labeled with “-”. If AriumMS is able to detect an analyte, it is labeled with “✓”, if not with “✗”. Analytes co-migrating with EOF are labeled with “*”. Analytes detected as co-migrating neutrals are labeled with “#”

### Non-target data evaluation

#### AriumMS workflow

AriumMS was developed as a universal and user-friendly computational metabolomics toolbox to tackle the challenge of multi-platform MS data analysis. The acronym AriumMS stands for augmented region of interest for untargeted metabolomics mass spectrometry. It was designed as a multi-tiered software for scalable (parallel processing of multiple sample sets) and reproducible data analysis. AriumMS consists of a main app (AriumMS, Fig. S2a) for ROI search, alignment, and low-level data filtering, and an evaluation App (AriumMSEval, Fig. S2b) for feature extraction and augmented data analysis. To ensure a high level of MS instrument compatibility, the open-source MS data format mzXML is used for raw data import [44]. To achieve optimal results in data processing, AriumMS uses user-defined sample groups. A sample group can contain datasets of different separation methods, different analytical workflows, or multiple phenotypes of biological samples. An individual parameterization can be applied for each group, for example, depending on the different peak characteristics of the separation methods used. Files are then batch-processed group by group. To reduce the computation time, a crop filter can be applied to discard areas of the measurements without relevant peaks. Several optional filters minimize the number of false-positive features during the ROI phase (e.g., isotope, adduct, and common contaminant filter) [45, 46]. An additional baseline correction removes any drift across the separation by baseline determination over a moving window by interpolation [47]. After processing the ROI stage, the data is automatically transferred to the AriumMSEval app. For automatic peak detection, the obtained ROIs are smoothed in the first step, and the difference between the smoothed ROI and the original ROI is used to estimate the noise level for this m/z. The second derivative of the smoothed ROI is formed, and peaks are identified by continuous wavelet transform (CWT) using the Mexican hat as the mother wavelet [48, 49]. Since real peaks are rarely perfectly symmetric, the peak boundaries are adjusted by a two-step process; first, via a friction border correction [50] based on the smoothed peak and then via moving standard deviation border correction based on the original peak. This algorithm can be found in the supplement information (Algorithm A1). Within the corrected limits, the peaks are now integrated, and the retention time is determined. Followed by the initial feature filtering of the AriumMSEval, possible feature filters are, for example, minimum and maximum peak width, minimum height within an ROI, and signal-to-noise ratio (supporting information Table S2). As an additional approach, an information entropy peak filter adapted from Ju et al. [51] was integrated. For a Gaussian peak, all points before the maximum have a constant positive slope, and after the maximum, a constant negative slope; these points are called normal points. Points that deviate from this condition are called variant points. Accordingly, the entropy of a peak can be expressed by the sum of the entropy of all possible events $H=-p*{log}_{2}\left(p\right)-q*{log}_{2}\left(q\right)$ [51, 52], where *p* is the number of variant points divided by the total number of points of the peak, and *q* is the number of normal points divided by the total number of points of the peak. *H* is calculated for each peak, and values greater than the median entropy are considered as noise and discarded. The peaks of the remaining features after the filtering were integrated, and the obtained areas are sorted into an *N* x *M* x *S* matrix, where *N* corresponds to the retention or migration times (rows), *M* corresponds to the m/z (columns), and *S* corresponds to the repeat measurement (layers). Based on the feature intensities obtained by the integration, the features can be scaled along the repeat measurements. Available scaling methods include center, auto, Pareto, vast, range, and level. Constant factors for whole groups and sample-specific factors can be applied as well. This allows, for example, normalization to the cell count of the sample or normalization to multiple internal standards for metabolite quantitation. Logarithmic and power transformations are available as well. A guideline for the selection of a proper scaling method is given by van den Berg et al. [53]. In the next step, the user-defined groups are augmented by linking the data cubes along the m/z dimension, combining the m/z and time dimensions into one dimension. The features are now named according to the following scheme: “m/z @Time, Group". If features of different groups have the identical mass and number of occurrences (no. of detections within the groups), they are assumed to be the same and labeled accordingly. The complete flow chart of the data processing can be found in Fig. S3.

#### AriumMS parameter screening and optimization

In order to obtain good results with the AriumMS software package, we applied an efficient D-optimal DOE for software parameter screening and optimization [54, 55]. The D-optimal DOE design enables the identification of optimal parameter settings with a lower number of required experiments compared to other designs. For that reason, the six repetitions of the metabolomics standard measured by all five analytical methods were evaluated regarding the number of found analytes and the total number of features. Found target features were defined by their m/z value and respective retention time (parameters for non-target data labeling are given in the supporting information). According to the results, a total of 126 target features were found. The total number of target features represents the number of target features detected by the five separation methods, including internal standards and co-migrating analytes. During the DOE screening, the ROI functions developed by Tauler [56] were tweaked by disabling the addition of random noise on the extracted ion chromatogram/electropherogram (EIC/E), since it was not required for AriumMS. By default, this algorithm added random noise on the EIC/E to remove possible gaps in the data. Here, the addition of random noise to the EIC/E created multiple peak tips and increased the peak splitting within the six repetitions of the standard, which induced varying retention times. Peak picking and integration of AriumMS were improved by the removal of the artificial distortion of the peak tips by the ROI function.

For an initial parameter screening, 14 parameters at two levels of both stages of the software (AriumMS, AriumMSEval) were chosen. The DOE identified the following parameters as significant for further optimization: ROI intensity threshold, mass spectra alignment, m/z error, and feature occurrence filter. The ROI intensity threshold defines the m/z intensity cutoff limit for noise. In general, a higher intensity threshold leads to a lower number of found features. For example, an increase of the ROI intensity threshold from 50,000 cts. to 150,000 cts. roughly loses 10% of total features/target features (HILIC ESI). As a universal robust ROI intensity threshold, we recommend 5–10% of the lowest base peak chromatogram/electropherogram (BPC/E) intensity. To ensure the comparability between repeated measurements for the same method and to counter the effects of analytical variance, the chromatograms could be alternatively aligned in time (recommended especially for CE) and m/z dimensions. The mass spectra alignment shifts measured masses to match the most common x quantile of detected masses (e.g., 0.95). Since it aligns masses, it offers benefits for QTOF instruments or for low-resolution MS. In general, resolution and calibration of the mass spectrometer must be considered for non-target data processing. Hence, the m/z error of the ROI should be set properly; here, 0.01 Da represents 10 ppm at 1000 Da (upper m/z limit). One of the most important feature filters of AriumMS is the minimum feature occurrence, which is defined as the relative minimum of feature detections per group. This filter leads to a reduction of the random noise within the MS data. If the minimum relative occurrence was set to 50%, the feature needs to appear in at least three out of six measurements. For higher confidence of the obtained features, higher percentages for the minimum relative occurrence were better. For example, the evaluation of the HILIC anion measurements showed 31 target features at 0% (≥ 1/6) occurrence, 30 target features at 50% (≥ 3/6) occurrence, and 23 target features at 100% (6/6) occurrence.

Within a D-optimal DOE optimization, further parameters were tested, and relevant parameters were optimized using three levels per parameter. These were minimum ROI size and minimum relative peak height. Minimum ROI size is defined as the minimum number of MS1 scans in which the m/z must be present in the EIC. This parameter is dependent on the processed separation method because the obtained feature peak width can differ between different separation methods Therefore, levels 5, 10, and 15 were tested. HILIC required higher ROI sizes (15, broader peaks) and smaller CE (< 10) because of the narrower peak width. In general, the minimum ROI size must be below the expected peak width of each separation method. The minimum relative peak height was significant for feature filtering (AriumMSEval). This filter analyzes each ROI and discards features below the relative peak height limit (%). A suitable value was 25%.

AriumMS offers the capability to define different parameter settings for the simultaneous processing of each evaluation group (different methods). Peak shapes and migration/retention time stability differ highly between CE and HILIC; therefore, minimum ROI group size and peak alignment (time) were probably the key aspects and should therefore be set for each group (method) individually. Especially, peak alignment becomes relevant for CE data due to migration time shifts that can occur between replicates (cp. avg. migration time deviation for CE [acidic BGE, anion]: ±0.9 min, and HILIC [ESI−]: ±0.1 min). The use of effective electrophoretic mobility instead of the migration time can address this issue [57] and will be implemented in AriumMS in the future.

#### Validation of the AriumMS feature generation

For the validation of the feature generation of AriumMS, the number of found targets and their respective integration were evaluated. The reliability of the data processing was tested with different file orders, and the required processing time of the overall workflow is given. Finally, the feature generation and peak integration algorithm of AriumMS was compared with the established universal open-source platform MZmine 3 [41]. For this validation, the MS data of all five analytical methods were processed with the optimized parameter settings (Supplement Table S2).

**Fig. 3:**
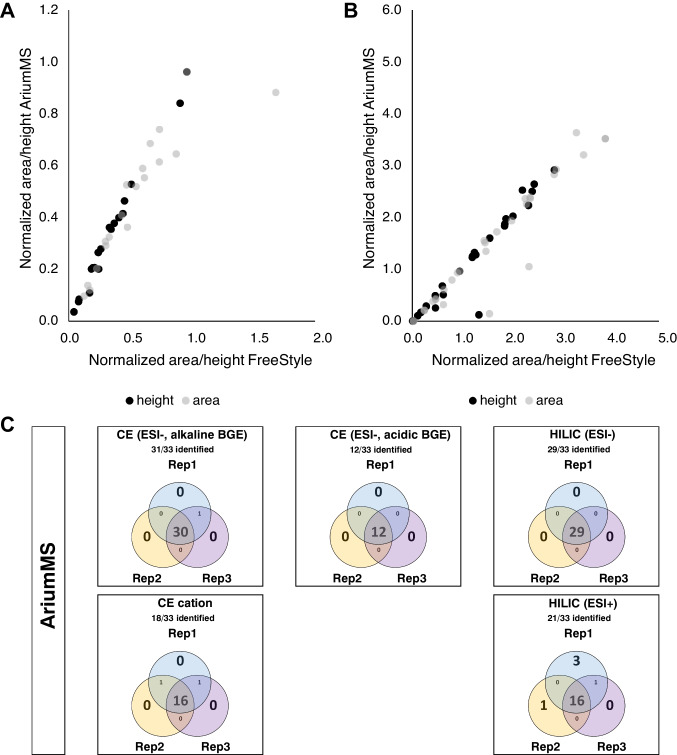
Comparison of peak integration algorithms of AriumMS using the example of CE-MS anion alkaline (**A**) and HILIC-MS anion (**B**). Evaluation of the independence of file order and simultaneously the repeatability of the peak finding in general for AriumMS (**C**). Venn diagrams are shown for each method

Using the optimized data processing settings, AriumMS was able to find 89% (112 of 126) of the target features of the standard at 50% occurrence level (features were detected in three of six measurements), as given in Table 1. For the evaluation of the AriumMS peak integration algorithm, two example datasets were chosen because of their different peak characteristics. These were CE-MS (alkaline BGE, anion) (Fig. 3a) and HILIC-MS (ESI−) (Fig. 3b). The peak heights and areas of AriumMS were compared with the results of the manual peak integration using FreeStyle, both normalized to an internal standard (supporting information). AriumMS was able to find 88% (CE) and 90% (HILIC) of the peak height and area compared to the manual integration. This finding can be explained by the function of the integration algorithm itself because the ROI intensity threshold is always subtracted from the peak. Furthermore, the peak integration of the HILIC method had two outliers compared to the manual integration, caused by limit cases of either non-baseline separated or very broad peaks and thus incorrect integration by the software. In general, the low deviation of the peak integration algorithm of AriumMS to the manual integration demonstrates the capabilities of this software tool for quantitation as generally requested for metabolomics tools [58].

To test the reliability of the data processing regarding the independence of the loaded file order and simultaneously the peak finding in general, the six data files of the repeat measurements were processed three times while only changing the order in which files were loaded. AriumMS generated similar results for all methods within the three file orders (Fig. 3c) except the HILIC cation. Here, three additional metabolites of the standard were found, caused by limit cases of either non-baseline separated analytes or very broad peaks.

AriumMS reduces the required data post-processing by the user significantly, compared to traditional metabolomics software, which is typically not optimized for multi-platform analytics. The processing of the whole dataset containing five analytical methods with six measurements takes about 60 min when AriumMS was used on a consumer-grade personal computer (PC) system with a 6-core CPU and 32 GB RAM). Considering the computation power of the computer used for AriumMS, an enterprise-grade computer (32-core CPU, 128 GB RAM) was able to reduce the required processing time to 50 min. Extensive data post-processing is not required for AriumMS due to its automated data augmentation of different methods (groups) and the integration of related statistical tools, which are mandatory for multi-platform data evaluation. For data evaluation, AriumMS contains advanced statistical evaluation tools such as labeling of false-positive features (false discovery rate, Benjamini–Hochberg procedure) [59], data scaling options (centering, Pareto, auto), transformation (power and log), and various plots (scatter plots, volcano plots, principal component analysis [PCA], and heatmaps). Because of the combination of the feature list generation and the statistical evaluation, no further data transfer into additional statistical software is required, which is an advantage compared to other metabolomics software. AriumMS is under active development, and the open-source code is continuously optimized to further improve the required data processing times.

In order to compare the feature generation and peak integration algorithm of AriumMS with the established universal open-source platform MZmine 3 [41], the data of the five analytical methods were processed with MZmine using optimized parameter settings and data processing options (Supplement Table S3). MZmine was able to find 87% (109 of 126 target features) and AriumMS found 89% (112 of 126 analytes) of the target features of the standard, both with an occurrence level of 50%. MZmine found 98% (CE) and 110% (HILIC) of the peak height and area compared to the manual integration. The comparison reveals that AriumMS and MZmine offer similar results regarding feature generation and peak integration, which highlights the solid foundation of the AriumMS feature extraction for augmented multi-platform data analysis.

### Augmented analytical workflows and data evaluation

#### Combination of different methods

**Fig. 4:**
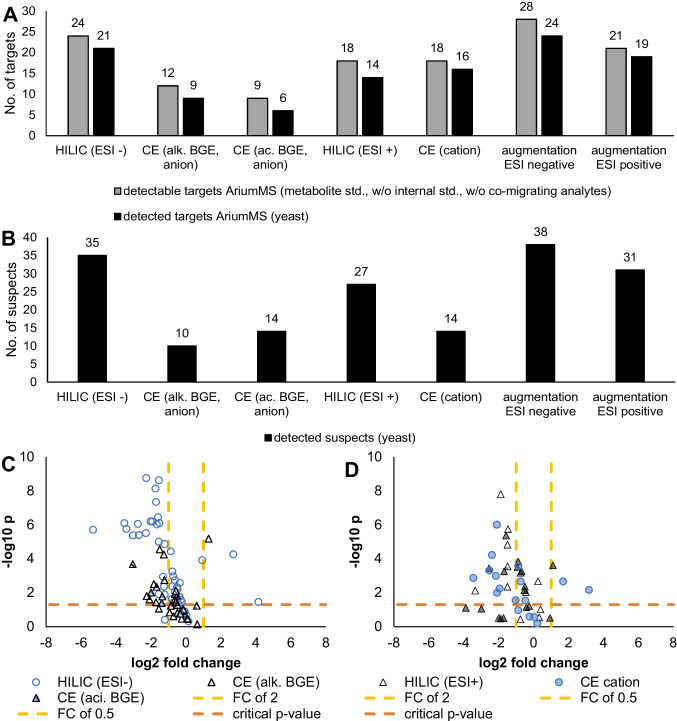
Results of the augmented data evaluation. **A** Shows the number of detectable standard metabolites by the methods itself and by mid-level augmented data evaluation using AriumMS presented with gray bars. The number of detected targets in the yeast extracts per method and by the mid-level augmented data evaluation using AriumMS in yeast extracts is presented with black bars. Both numbers are without internal standards and co-migrating analytes. **B** Shows the number of found suspects per method and augmentation. Volcano plots for the two augmentations are presented in (**C**) ESI negative and (**D**) ESI positive mode

The combination of multiple analytical methods—here, CE-MS and HILIC-MS—increases the feature capacity by their different selectivity. Each separation technique (either HILIC or CE) was not able to detect all 36 metabolites of the standard (Fig. 4a, Table 1). The multi-platform data evaluation offers the possibility to increase the analytical coverage of a non-target metabolomics workflow. In an environment as complex as metabolomics samples, the number of detectable features (target, suspect, non-target) can be expected to be higher. Hence, two combinations for the augmented analysis were tested. Based on the AriumMS evaluation of the standard, the mid-level augmented data evaluation of all three ESI negative methods enables the coverage of up to 30 target metabolites (w/o co-migrating metabolites). Combining the two ESI positive methods allows the coverage of up to 23 target metabolites.

#### Application: yeast metabolome

As a case study, two yeast CEN.PK122 [42] cell cultures were analyzed, one as reference (Mock) and the other treated with halogenated indole (Effect1). Adding halogenated indole to yeast resulted in a clearly decreased growth rate, as shown in Fig. S1. The induced effect on the yeast metabolome was analyzed here by the five different analytical methods and augmented evaluation by AriumMS. In principle, multi-platform metabolomics offers two major improvements. Firstly, it is possible to increase the analytical metabolome coverage, and secondly, observed metabolic effects are cross-evaluated by another method (if detected in both). Generally, the data evaluation here was based on three levels. Starting with a target evaluation, based on the feature list obtained by AriumMS, target features were assigned by their m/z value and RT/MT compared to the reference values of the metabolite standard, followed by a suspect evaluation, where target features were assigned by m/z values of a suspect database. Moreover, in a non-targeted evaluation, the overall feature lists of Mock and Effect1 were compared.

The mid-level augmented data evaluation shows that the combination of all ESI negative methods (HILIC [ESI−], CE alkaline BGE, CE acidic BGE) detects 24 targets. The ESI positive augmentation (HILIC [ESI+, CE cation]) detects 19 targets (Table 2, Fig. 4a). If just one method is applied for the metabolome analysis, the number of found features is decreased because HILIC (ESI+) detects just 14, and CE (cation) 16 targets. Figure 4a shows a comparison of target numbers found by each method and by the augmentation. The total number of detectable targets in the yeast extracts was lower than in the metabolite standard due to their absence in the yeast metabolism (Table 2 “not present”) or low biological concentration. The respective number of detectable target metabolites was reduced to 28 for ESI negative augmentation and 21 for ESI positive augmentation (Fig. 4a).

**Table 2:** Results of the augmented multi-platform data analysis of yeast extracts based on targets

Group	Analyte		ESI negative augmentation		ESI positive augmentation	
	ID	Name	[M−H]^−^	HILIC/CE (alk.)/CE (aci.)	[M+H]^+^	HILIC/CE
Amino acid	1	L-serine	104.0348	down / down* / -	106.0504	n.a. / down
	2	L-proline	114.0555	down / stable* / -	116.0712	stable / down
	3	L-valine	116.0712	stable / stable* / -	118.0869	stable / stable
	4	L-threonine	118.0504	down / stable* / -	120.0661	down / stable
	5	L-cysteine	120.0119	- / n.a.* / -	122.0276	n.a. / n.a.
	6	L-leucine	130.0869	stable / stable* / -	132.1025	knock out / stable
	7	L-isoleucine	130.0869	down / n.a.* / -	132.1025	n.a. / induced
	8	L-aspartic acid	132.0297	down / stable / n.a.	134.0454	down / induced
	9	L-lysine	145.0978	- / stable / -	147.1134	n.a. / stable
	10	L-glutamic acid	146.0454	stable / stable / -	148.0610	stable / stable
	11	L-methionine	148.0433	down / knock out* / -	150.0589	down / down
	12	L-histidine	154.0617	- / down / -	156.0773	- / stable
	13	L-phenylalanine	164.0712	stable / up* / n.a.	166.0869	stable / stable
	14	L-arginine	173.1039	- / stable / -	175.1196	- / up
	15	L-tyrosine	180.0661	stable / stable* / -	182.0818	down / induced
	16	L-tryptophan	203.0821	knock out / knock out* / -	205.0978	n.a. / knock out
Internal std	17	benzenesulfinic acid	141.0010	**used for internal standardization**	143.0167	**used for internal standardization**
	18	2-nitrobenzoic acid	166.0140		168.0297	
	19	methionine sulfone	180.0331		182.0487	
	20	pentetic acid	392.1306		394.1462	
Metabolites	21	succinate	117.0188	down /—/ n.a.	119.0345	- / -
	22	nicotinic acid	122.0242	stable / stable / stable	124.0399	stable / knock out
	23	tartaric acid	149.0086	- /—/ n.a.	151.0243	- / -
	24	citrate; citric acid	191.0192	- /—/ n.a.	193.0348	- / -
	25	caffeine	193.0726	not present	195.0882	not present
	26	ATP	505.9879	n.a. / n.a. / -	508.0036	stable / -
	27	NAD	662.1013	down / stable / n.a.	664.1170	stable / -
	28	NADH	664.1170	n.a. / n.a. / -	666.1327	knock out / -
Carbohydrates	29	L-fuc., 6-deoxy-L-gal	163.0607	- / n.a.* / n.a.*	165.0763	- / -
	30	D-mannose	179.0556	knock out / knock out* / n.a.*	181.0713	- / -
	31	α-D-glucose	179.0556	n.a. / n.a.* / n.a.*	181.0713	- / -
	32	α-D-galactose	179.0556	n.a. / n.a.* / n.a.*	181.0713	- / -
	33	D-mannose-1-PO_4_	259.0219	stable / stable/ stable	261.0376	knock out / -
	34	β-D-fructose-6-PO_4_	259.0219	down / n.a. / stable	261.0376	n.a. / -
	35	α-D-glucose 1-PO_4_	259.0219	down / n.a. / stable	261.0376	n.a. / -
	36	α-D-galactose-1-PO_4_	259.0219	stable / stable / stable	261.0376	n.a. / -
	37	N-acetylneuraminate	307.0909	not present	309.1066	
	38	UDP-glucose	565.0472	stable / n.a. / knock out	567.0629	n.a. / -
	39	GDP-L-fucose	588.0744	not present	590.0901	not present
	40	CMP-N-acetylneuraminate	613.1395	not present	615.1552	not present
Number of detected targets (w/o co-migrating analytes)			anion	24	cation	19

Regulation calculated by the FC (indole-treated/reference): FC < 0.50, down; 0.50 ≤ FC ≤ 2.0, stable; FC ≥ 2.00, up. Non-detected analytes are labeled as “n.a.”, undetectable analytes are labeled as “-”. Knocked-out analytes by halogenated indole treatment are labeled as “knock out,” and newly occurring analytes by treatment are labeled as “induced.” Co-migrating analytes are labeled with *. The order of the presented results is as follows: (i) augmentation ESI negative: HILIC (ESI−)/CE (alkaline, anion)/CE (acidic, anion), and (ii) augmentation ESI positive: HILIC (ESI+)/CE (cation)

For the suspect evaluation, metabolites of the glycolysis, gluconeogenesis, TCA (tricarboxylic acid) cycle, and amino acid metabolism were analytes of interest. Therefore, a list containing the m/z values of relevant metabolites (Table S4) was used for a m/z search and labeling within the generated feature lists of the measured yeast extracts (labeling within a ± 0.03 m/z range). Again, the multi-platform analysis offers a higher analytical metabolome coverage than single methods, as shown in Fig. 4b and supplement Table S4. In addition, two mutually confirming analytical methods lead to higher confidence in suspect feature search only based on m/z values. Here, the ESI negative augmentation finds 38 suspects, and the ESI positive 31 suspects (Fig. 4b). The three CE methods offer the capability to cross-prove several of the observed suspect effects by the HILIC methods (Table S4). Most of the suspect regulation fold changes matched between the methods; a reason for varying results could be the similar m/z values of different metabolites or different matrix effects (for example, co-migration/elution of metabolites or BGE effects). This issue can be addressed with the implementation of the MS/MS confirmation and will be implemented soon in AriumMS and a database search. Most of the targets and suspects were significantly downregulated between Mock and Effect1, shown in Fig. 4c. An observed metabolic effect of the ESI negative augmentation was the change in sugar metabolism (Table 2, supplement Table S4, Fig. S4). The sum parameter of mannose-1-phosphate and galactose-1-phosphate showed a slight downregulation with fold change (FC) of 0.9 (HILIC ESI−, indole-treated effect divided by reference cell effect). The sum parameter of fructose-6-phosphate and glucose-1-phosphate was even more downregulated (FC 0.3, HILIC ESI−, Fig. S4 e–f). The CE-MS method (alkaline BGE) showed the same downregulation of glucose-1-phosphate (Fig. S4a–b). On the suspect level, the appearance of two additional disaccharides was observed (Fig. S4g–h). Hence, indole treatment may lead to lower levels of glucose, hindering the production of mannose-1-phosphate and glucose-6-phosphate, causing increased production of lactose and saccharose. Lactose cannot be digested by yeast cells because of the lacking lactase enzyme. Furthermore, within the two augmentations, L-tryptophan was knocked out in the indole-treated samples (Effect1).

For the non-target evaluation, three PCAs were conducted, which contain the features of Mock and Effect1 (feature occurrence ≥ 50%). Each of the three PCAs was a combination of an HILIC (ESI±) method with one CE method. Figure S5 shows that the measured samples (Mock and Effect1) were partitioned into two major groups derived by the treatment with halogenated indole with at least 79.5% of the explained variance on the first principal component (PC1). As a result, the treatment with halogenated indole has a strong influence on the yeast metabolome. The integration of several LC-MS/CE-MS techniques expands metabolome coverage and increases the confidence of the metabolic results. This makes AriumMS a powerful tool for multi-platform metabolomics.

## Conclusions

Multi-platform metabolomics by high-resolution MS based on several orthogonal separation mechanisms of CE and LC maximizes the metabolome coverage. The AriumMS software toolbox presented here is a powerful tool for fast untargeted processing of these augmented datasets. AriumMS contains ROI search, preprocessing, feature detection and integration, false-positive filter, scaling, and transformation followed by the augmentation and various chemometric data evaluation tools. The validation of the feature detection and mid-level fusion steps were successfully performed using a multi-analyte standard. In AriumMS all processing steps were integrated into a single user-friendly software tool at a high level of flexibility and automatization. Further developments will include the implementation of MS/MS spectral networking in order to precisely and autonomously connect features detected by two or more methods. Furthermore, the augmentation of spectroscopic data with chromatographic/electrophoretic data might be interesting.

The AriumMS tool presented here was used to process datasets obtained from HILIC-MS and CE-MS measurements of a multi-analyte standard and yeast extracts. All CE-MS methods show in general narrow peaks and benefit from the sensitivity of the nanoCEasy interface. Additionally, the valving functionality of the nanoCEasy interface enables capillary reconditioning between the injections. HILIC-MS and CE-MS show a large overlap with only a few analytes detected by only one of the methods. HILIC-MS also covers neutral polar analytes, such as carbohydrates, whereas the basic BGE in particular allows the separation of various isomeric anions such as hexose phosphates by CE-MS.

AriumMS was successfully applied for mid-level data fusion to remove irrelevant information such as artifacts and noise from the entire analytical dataset (LC-MS + CE-MS) of yeast extracts. The results confirm the great advantage of flexible parameterization for processing of individual separation methods with different peak characteristics. Multi-platform metabolomics expands metabolome coverage and increases the confidence of the metabolic results.

## Supplementary Information

Below is the link to the electronic supplementary material.

Supplementary file1 (DOCX 1.51 MB)

## Data Availability

The datasets generated during and/or analyzed during the current study are available from the corresponding author on reasonable request. AriumMS 1.0.0 is available at GitHub: https://github.com/AdrianHaun/AriumMS/

## Abbreviations

A.u.: Arbitrary units
AriumMS: Augmented region of interest for untargeted metabolomics mass spectrometry
BGE: Background electrolytes
BPC/E: Base peak chromatogram/electropherogram
CE: Capillary electrophoresis
CWT: Continuous wavelet transform
DMSO: Dimethyl sulfoxide
DOE: Design of experiment
EIC/E: Extracted ion chromatogram/electropherogram
EOF: Electro-osmotic flow
ESI: Electrospray ionization
FC: Fold change
GC: Gas chromatography
HCD: Higher-energy collisional dissociation
HILIC: Hydrophilic interaction liquid chromatography
MS: Mass spectrometry
NAD: Nicotinamide adenine dinucleotide
PCA: Principal component analysis
PC: Principal component
QTOF: Quadrupole time of flight
ROI: Region of interest
RP-LC: Reversed-phase liquid chromatography
sd: Standard deviation
SD: Synthetic dextrose minimal medium
SL: Sheath liquid
