# Supplementary material for: Augmented region of interest for untargeted metabolomics mass spectrometry (AriumMS) of multi-platform-based CE-MS and LC-MS data
Source: Anal Bioanal Chem. 2023 May 25;415(16):3137–54. doi: 10.1007/s00216-023-04715-6 (PMC10287804; doi:10.1007/s00216-023-04715-6)
Supplement: Supplementary file 1 — Supplementary file1 (DOCX 1.51 MB) [file 216_2023_4715_MOESM1_ESM.docx]

**Supporting Information**

**Augmented region of interest for untargeted metabolomics mass spectrometry (AriumMS) of multiplatform-based CE-MS and LC-MS data**

Lukas Naumann^1‡^, Adrian Haun^1‡^, Alisa Höchsmann^1^, Michael Mohr^1^, Martin Novák^1^, Dirk Flottmann^1^, Christian Neusüß^1^*

^1^ Department of Chemistry, Aalen University, Beethovenstraße 1, 73430 Aalen, Germany

^‡^These authors contributed equally.

**Corresponding Author**

*E-Mail: Christian.Neusuess@hs-aalen.de; phone: +49 7361 576-2399

**Table of contents**

- Materials & Methods
  - Additional information
  - Supplement Table S1: MSConvert parameter
  - Supplement Table S2: *AriumMS* parameter
  - Supplement Table S3: MZmine parameter
  - Algorithm 1: Moving Standard Deviation Border Correction Algorithm
- Evaluation of the analytical methods
  - Hydrophilic interaction liquid chromatography-mass spectrometry
  - Capillary electrophoresis - mass spectrometry
- Tables:
  - Supplement Table S4: Yeast suspect evaluation
- Figures:
  - Fig. S1: Yeast growth
  - Fig. S2: *AriumMS* GUI
  - Fig. S3: *AriumMS* processing workflow
  - Fig. S4: Yeast metabolomics, observed effects
  - Fig. S5: PCA of yeast extracts

# Materials & Method (additional information)

## Materials

Ultrapure water (<0.057 µs/cm, SG water purification system Ultra Clear; Siemens Water Technologies, Günzburg, Germany) was used to prepare all eluents, background electrolytes (BGE), sheath liquids (SL), water-based solutions, and rinsing solutions. Acetonitrile, methanol, 2-propanol (all solvents LC-MS Grade, >99.95 %, ROTISOLV®), formic acid (FAc; p.a., >98 %), hydrochloric acid (HCl; p.a., >98 %, all acids ROTIPURAN®), chloroform (HPLC grade, ROTISOLV®), ammonium acetate (≥ 97%, p.a.), and sodium hydroxide (NaOH; >99 %) were obtained from Carl Roth (Karlsruhe, Germany). Hydrofluoric acid (HF; p.a. ≥38 %) was purchased from Sigma Aldrich (Darmstadt, Germany). Pierce™ FlexMix™ calibration solution was purchased from Thermo Fisher Scientific (Rockford, IL, USA). The amino acid standard (1 nmol/µL in 0,1 M HCl) is obtained from Agilent (Agilent Technologies, Santa Clara, CA, US). The internal standards and metabolites used are obtained from Sigma-Aldrich (St. Louis, MO, USA). Sugars (nucleotide sugars, phosphate sugars) are purchased from Biosynth Carbosynth (Staad, Switzerland). Synthetic Dextrose Minimal Medium (SD, synthetic minimal medium) obtained from Carl Roth (Karlsruhe, Germany). Glass beads (acid-washed, 425-600 μM, Sigma Aldrich, St. Louis, MO, US), Ultrafree®-MC GV centrifugal filters 0.22 μM (Merck Millipore, Burlington, MA, US).

## Metabolite standard

Metabolite standard consists of 41 pure substances diluted with purified water (origin of substances given in the materials section). The standard is pipetted together out of 4 stock solutions with a concentration of 1 mM: internal standards (4), yeast metabolites (8), amino acids (16), sugars (nucleotide sugars, hexose-phosphates, hexoses) (12). The used standard concentrations of the mixture are 100 and 25 µM. The 100 µM concentration was used for all evaluations of this publication. The compounds are the following: internal standards: benzene sulfonic acid, 2-nitrobenzoic acid, methionine sulfone, pentetic acid; metabolites: succinic acid, nicotinic acid, tartaric acid, citric acid, caffeine, ATP, NAD, NADH; amino acids: L-serine, L-proline, L-valine, L-threonine, L-cysteine, L-leucine, L-isoleucine, L-aspartic acid, L-lysine, L-glutamic acid, L-methionine, L-histidine, L-phenylalanine, L-arginine, L-tyrosine, L-tryptophan; sugars: L-fucose, D-glucose, D-mannose, D-galactose, α-D-glucose-1-phosphate, D-fructose-6-phosphate, UDP-D-glucose, N-acetylneuraminic-acid, α-D-galactose-1-phosphate, α-D-mannose-1-phosphate, CMP-Neu5Ac, GDP-L-fucose, GDP-D-mannose.

## Metabolomics preparation

The lyophilized cells (8 mg) were dissolved in 485 µL ice-cold methanol/purified water (50/50, v/v) + 15 µL internal standard stock solution (1 mM), and disrupted with glass beads. After 15 min centrifugation, the non-soluble cellular components were removed by ultrafiltration. Lipids were removed using liquid-liquid extraction, by adding ice-cold 350 µL chloroform to the flow-through of the ultrafiltration. After vortexing and a 10 min storage on ice, the sample was centrifuged and the non-polar chloroform phase was discarded, and the aqueous phase dried within a speedvac. The dried sample was dissolved in 100 µL purified water.

## Data evaluation

**Data acquisition and ion trace extraction (EIC).** Data acquisition was performed with Orbitrap, controlled by Thermo Scientific Xcalibur 4.1.50 and Orbitrap Tribrid MS Series Instrument Control Software Version 3.2 (Thermo Fisher Scientific, San Jose CA, USA). Extraction of ion traces was done with FreeStyle 1.5.93.34 (Thermo Fisher Scientific, San Jose CA, USA). Used m/z values are given in **Table 1**. Automated EIC evaluation was performed in Microsoft Excel 2016 (Microsoft Corporation, Redmond WA, USA).

**Non-target data evaluation utilizing *AriumMS*.**

*ArumMS* version 1.0 was used (https://github.com/AdrianHaun/AriumMS/; Adrian.Haun@hs-aalen.de) for non-target data evaluation and augmentation. *AriumMS* is based on Matlab (MathWorks, Natick MA, USA) and uses the following software packages and functions:

- Bioinformatics Toolbox: *mzxmlinfo*, *mzxmlread*, *mzxml2peaks,*, *msalign, msbackadj*, *mssgolay (*https://www.mathworks.com/products/bioinfo.html)
- Statistics and Machine Learning Toolbox:

*gscatter, biplot, ttest2, pca* (https://www.mathworks.com/products/statistics.html)

- Signal Processing Toolbox:

*findpeaks (*https://www.mathworks.com/products/signal.html)

- Image Processing Toolbox:

*imextendedmax, imextendedmin* (https://www.mathworks.com/products/image.html)

- Wavelet Toolbox:

*cwtfilterbank, wt* (https://www.mathworks.com/products/wavelet.html)

- Parallel Computing Toolbox:

*parfor (*https://www.mathworks.com/products/parallel-computing.html)

For initial data conversion *MSconvert* *3* (ProteoWizard, Palo Alto CA, USA, [1]) is used. Parameters are given in **Table S1**. The final set of parameters for non-target data processing and augmentation using *AriumMS* are given in **Table S2**. The repeat measurements of each analytical method were selected and simultaneously processed in groups. The ROI and preprocessing steps were chosen individually for each group. Post-data evaluation of the feature lists was performed in Microsoft Excel 2016 (Microsoft Corporation, Redmond WA, USA).

**Non-target data evaluation utilizing *MZmine*.**

For *MZmine* 3.2.3 [2] mzXML data were used (Table S1). The final set of parameters for non-target data processing and augmentation using *MZmine* *3* are given in **Table S3**. Post-data evaluation of the feature lists was performed in Microsoft Excel 2016 (Microsoft Corporation, Redmond WA, USA).

**Non-target data annotation**

Feature annotation was performed with a self-written Excel macro sheet. Found analytes are annotated by a comparison of m/z (± 0.03 Da annotation range) and RT/MT (CE; ± 5 min, LC: ± 1.5 min) to a local database. Suspect features are annotated by a comparison of m/z (± 0.03 Da annotation range) to a local database.

**Evaluation of Software integration algorithm.**

Identified targets from feature lists (*AriumMS*) were normalized to the selected internal standard (CE alkaline anion: 2-nitrobenzoic acid, HILIC (ESI -): benzene sulfinic acid). The same goes for the manually integrated Targets using FreeStyle. For evaluation normalized target intensities (or areas) of *AriumMS* are subtracted by the normalized target intensities (or areas) of the manual integration.

**Table S1:** Orbitrap MS RAW data preprocessing for data analysis by *MSConvert* (ProteoWizard)

| **category** | **parameter** | **settings** | | | | |
| --- | --- | --- | --- | --- | --- | --- |
|  |  | **CE alkaline BGE, anion** | **CE acidic BGE, anion** | **CE**  **cation** | **HILIC**  **(ESI -)** | **HILIC**  **(ESI +)** |
| Options | Output format | mzXML | | | | |
|  | Extension | - | | | | |
|  | Binary encoding precision | 32-bit | | | | |
|  | Write index | used | | | | |
|  | Use zlib compression | not used | | | | |
|  | TPP compatibility | used | | | | |
|  | Package in gzip | not used | | | | |
|  | Combine ion mobility scans | not used | | | | |
|  | SIM as spectra | not used | | | | |
|  | SRM as spectra | not used | | | | |
| Presets |  | generic defaults | | | | |
| Filters | Subset | not used | | | | |

**Table S2:** Chosen parameter & settings for *AriumMS* data analysis. If parameter setting is used for all groups value is written only once.

|  | **category** | **parameter** | **settings** | | | | |
| --- | --- | --- | --- | --- | --- | --- | --- |
|  |  |  | **CE alkaline BGE, anion** | **CE acidic BGE, anion** | **CE**  **cation** | **HILIC (ESI -)** | **HILIC (ESI +)** |
| ***AriumMS*** | Number of groups | | 5 | | | | |
|  | main options | MS polarity | - | - | + | - | + |
|  |  | ISTD normalization | No | | | | |
|  |  | Blank Subtraction | No | | | | |
|  |  | Alignment | Yes | | | | |
|  |  | Baseline Correction | Yes | | | | |
|  |  | Golay Smoothing | Yes | | | | |
|  |  | Isotope Filter | No | | | | |
|  |  | Adduct Filter | No | | | | |
|  |  | Contaminant filter | No | | | | |
|  | ROI parameters | Intensity threshold | 5.0E4 | | | | |
|  |  | MZ error [Da] | 0.01 | | | | |
|  |  | Min ROI size | 10 | 5 | 10 | 15 | 20 |
|  |  | Start [s] | 0 | | | | |
|  |  | End [s] | 1802 | 2434 | 1602 | 1500 | 1500 |
|  | Alignment parameters | Mass spectra Alignment | No | | | | |
|  |  | Quantile value | - | | | | |
|  |  | Estimation method | - | | | | |
|  |  | Correction method | - | | | | |
|  |  | Peak alignment | Yes | | | | |
|  |  | Maximum shift [scans] | 700 | 700 | 400 | 100 | 400 |
|  |  | Pulse width [scans] | 5 | 5 | 15 | 15 | 5 |
|  |  | Window size ratio | 2.5 | | | | |
|  |  | Search space | Evenly Spaced Lattice | | | | |
|  |  | Iterations | 4 | 4 | 4 | 7 | 7 |
|  |  | Grid steps | 20 | | | | |
|  | Baseline correction parameters | Shifting window size | 200 | | | | |
|  |  | Step size | 200 | | | | |
|  |  | Regression method | Shape-preserving piecewise cubic interpolation | | | | |
|  |  | Estimation method | Quantile | | | | |
|  |  | Smoothing method | None | | | | |
|  |  | Quantile value | 0.1 | | | | |
|  | Golay smoothing parameter | Frame size | 15 | | | | |
|  |  | Polynomial degree | 2 | | | | |
| ***AriumMSEval*** | ISTD processing options | ISTD normalization | - | | | | |
|  |  | Use ISTD as mass cal. | - | | | | |
|  |  | Processing stage | - | | | | |
|  | Data Pre-Treatment | Scaling options | - | | | | |
|  |  | Transformations | - | | | | |
|  | Feature Sorting parameters | m/z tolerance [Da] | 0.01 | | | | |
|  |  | RT tolerance [s] | 10 | | | | |
|  | Define sample-specific scaling factors | | - | | | | |
|  | Feature extraction | Select eval. Parameter | Peak height and area used | | | | |
|  | Feature filter | Min peak height [%] | 25 | | | | |
|  |  | Min peak width [s] | 4 | | | | |
|  |  | Max peak width [s] | 120 | | | | |
|  |  | Min S/N | 10 | | | | |
|  |  | Min occ. per group [%] | 50 | | | | |
|  |  | Peak entropy filter | Yes | | | | |
|  |  | Benjamini Hochberg filter | No | | | | |
|  |  | False discovery rate [%] | - | | | | |
|  |  | p-value | 0.05 | | | | |
|  |  | Fold change | 2 | | | | |

**Table S3:** Chosen parameter & settings for *MZmine* data analysis

| **category** | **parameter** | **settings** | | | | |
| --- | --- | --- | --- | --- | --- | --- |
|  |  | **CE alkaline BGE, anion** | **CE acidic BGE, anion** | **CE**  **cation** | **HILIC**  **(ESI -)** | **HILIC**  **(ESI +)** |
| mass detection | Retention time | Auto R. | Auto R. | Auto R. | Auto R. | Auto R. |
|  | MS level | 1 | 1 | 1 | 1 | 1 |
|  | Mass detector | Exact mass | Exact mass | Exact mass | Exact mass | Exact mass |
|  | Noise level | 5.0E4 | 5.0E4 | 5.0E4 | 5.0E4 | 5.0E4 |
|  | Output netCDF filename (optional) | - | - | - | - | - |
| ADAP chromatogram builder | retention time | Auto | auto | auto | auto | auto |
|  | MS level | 1 | 1 | 1 | 1 | 1 |
|  | Min group size in # of scans | 5 | 5 | 5 | 5 | 5 |
|  | Group intensity threshold | 1.0E5 | 1.0E5 | 1.0E5 | 1.0E5 | 1.0E5 |
|  | Min highest intensity | 5.0E4 | 5.0E4 | 5.0E4 | 5.0E4 | 5.0E4 |
|  | m/z tolerance [ppm] | 10 | 10 | 10 | 10 | 10 |
| Baseline correction | Chromatogram type | BPI | BPI | BPI | BPI | BPI |
|  | MS Level | 1 | 1 | 1 | 1 | 1 |
|  | m/z bin width | 1 | 1 | 1 | 1 | 1 |
|  | Correction Method | asymmetric | asymmetric | asymmetric | asymmetric | asymmetric |
|  | R engine | RCaller | RCaller | RCaller | RCaller | RCaller |
| Savitzky Golay smoothing | Filter width | 5 | 5 | 5 | 5 | 5 |
| ADAP feature resolver | Algorithm | ADAP | ADAP | ADAP | ADAP | ADAP |
|  | S/N threshold | 10 | 10 | 10 | 10 | 10 |
|  | S/N estimator | Int win SN | Int win SN | Int win SN | Int win SN | Int win SN |
|  | min feature height | 1E5 | 1E5 | 1E5 | 1E5 | 1E5 |
|  | Coefficient/ area threshold | 100 | 100 | 100 | 100 | 100 |
|  | Peak duration range | 0.0-.2.0 | 0.0-.2.0 | 0.0-.2.0 | 0.0-.2.0 | 0.0-.2.0 |
|  | RT wavelet range | 0.0-0.3 | 0.0-0.3 | 0.0-0.3 | 0.0-0.3 | 0.0-0.3 |
|  | m/z center calculation | AUTO | AUTO | AUTO | AUTO | AUTO |
|  | m/z range for MS2 scan pairing [Da] | - | - | - | - | - |
|  | RT range for MS2 scan pairing [min] | - | - | - | - | - |
| 13C isotope filter | m/z tolerance [m/z, ppm] | 0.001, 5 | 0.001, 5 | 0.001, 5 | 0.001, 5 | 0.001, 5 |
|  | Retention time tolerance | 0.02 | 0.02 | 0.02 | 0.02 | 0.02 |
|  | Mono-tonic shape | + | + | + | + | + |
|  | Maximum charge | 3 | 3 | 3 | 3 | 3 |
|  | Representative isotope | most int. | most int. | most int. | most int. | most int. |
| Local minimum feature resolver | Chromatographic threshold [%] | 70 | 70 | 70 | 70 | 70 |
|  | Minimum search range (RT) | 0.05 | 0.05 | 0.05 | 0.05 | 0.05 |
|  | Minimum relative height [%] | 15 | 15 | 15 | 15 | 15 |
|  | Minimum absolute height | 1E5 | 1E5 | 1E5 | 1E5 | 1E5 |
|  | Min ratio of peak top/edge | 1.7 | 1.7 | 1.7 | 1.7 | 1.7 |
|  | Peak duration range | 0.0-2.0 | 0.0-2.0 | 0.0-2.0 | 0.0-2.0 | 0.0-2.0 |
|  | Min # of data points | 5 | 5 | 5 | 5 | 5 |
| RANSAC aligner | m/z Tolerance [ppm] | 10 | 10 | 10 | 10 | 10 |
|  | RT tolerance [min] | 2 | 2 | 2 | 2 | 2 |
|  | RT tolerance after correction [min] | 0.25 | 0.25 | 0.25 | 0.25 | 0.25 |
|  | RANSAC iterations | 6 | 6 | 6 | 6 | 6 |
|  | Minimum number of points [%] | 60 | 60 | 60 | 60 | 60 |
|  | Threshold value | 1 | 1 | 1 | 1 | 1 |
|  | Linear model | - | - | - | - | - |
|  | Require same charge state | - | - | - | - | - |

**Table S3 (continued):** Chosen parameter & settings for *MZmine* data analysis

| **category** | **parameter** | **settings** | | | | |
| --- | --- | --- | --- | --- | --- | --- |
|  |  | **CE alkaline BGE, anion** | **CE acidic BGE, anion** | **CE**  **cation** | **HILIC**  **(ESI -)** | **HILIC**  **(ESI +)** |
| MS/MS spectral networking (Online Databank annotation) | m/z tolerance [ppm] | 10 | 10 | 10 | 10 | 10 |
|  | Min height | 0E0 | 0E0 | 0E0 | 0E0 | 0E0 |
|  | Minimum data points | 3 | 3 | 3 | 3 | 3 |
|  | Minimum matched signals | 3 | 3 | 3 | 3 | 3 |
|  | Min cosine similarity | 0.7 | 0.7 | 0.7 | 0.7 | 0.7 |
|  | Modification aware similarity | + | + | + | + | + |
|  | Check MS2 neutral loss similarity | - | - | - | - | - |
| Custom database Search | Field separator | ; | ; | ; | ; | ; |
|  | m/z | + | + | + | + | + |
|  | RT | + | + | + | + | + |
|  | name | + | + | + | + | + |
|  | adduct | M-H^-^ and M-2H^2-^ | M-H^-^ and M-2H^2-^ | M+H^+^ and M+NH4^+^ | M-H^-^ and M-2H^2-^ | M+H^+^ and M+NH4^+^ |
|  | m/z Tolerance [ppm] | 10 | 10 | 10 | 10 | 10 |
|  | RT tolerance [min] | 3 | 3 | 3 | 1 | 1 |
|  | CCS tolerance [%] | 5 | 5 | 5 | 5 | 5 |

Moving Standard Deviation Border Correction Algorithm

Since the friction border correction is applied to the smoothed peaks, the peak boundaries slip too far. The second correction is applied to move the borders back toward the peak. The detection of a peak is done by comparing the difference between the next intensity and the average intensity in this range, and the standard deviation of the range. For this step, the original non-smoothed peaks are used.

**Algorithm A1:** Moving Standard Deviation Border Correction Function numel returns the number of elements in an array, mean calculates the mean of an array and std calculates the Standard deviation of an array

| Input: *Y*: Chromatogram Data  Input: *p*: List of peak boundary pairs  1: **for** each (*u,l*) ∈ *p*  2: **while** *u* > 1 ∧ *u* < numel(*Y*) – 8 **do**  3: **if** *Y*_u-1_ - mean(Y_u-1:u+7_) ≤ std(*Y*_u-1:u+7_) ∨ *Y*_u-1_-*Y*_u_ = 0 **then**  4: *u* ← *u* - 1  5: **while** l ≤ numel(*Y*)-1 ∧ *l* > 8 **do**  6: **if** *Y_l_*_+1_ - mean(Y*_l_*_-7:_*_l_*_+1_) ≤ std(Y*_l_*_-7:_*_l_*_+1_) ∨ Y*_l_*-Y*_l_*_+1_ = 0 **then**  7: *l* ← *l* + 1 |
| --- |

# Evaluation of the analytical methods

## Hydrophilic interaction liquid chromatography-mass spectrometry

A simple HILIC gradient was combined with either ESI-MS in positive (+) or in negative (-) ion mode. The gradient was optimized regarding stable retention times, short run times, and low MS contamination. Stable retention times of ± 0.1 min (average of all analytes) were achieved by a 10 min equilibration time after each run (**Table 1**). The base peak chromatograms (BPC) are shown in **Fig. 2a** (anionic) and **Fig. 2d** (cationic). The anion HILIC-MS method (ESI -) was able to detect 27 out of 36 substances of the metabolomics standard mix. The cationic method (ESI +) could detect 25 substances of the metabolomics standard mix. HILIC-MS (ESI +) was able to partially separate the three hexoses of the standard, whereas the four hexose-phosphates could not be separated. The isomeric amino acids L-leucine and L-isoleucine were baseline separated and detectable with both ESI modes of the HILIC-MS method.

## Capillary electrophoresis - mass spectrometry

The cationic CE-MS method is based on an acidic BGE and is highly suitable for the separation of cations, i.e. containing an amino group. This method was capable to detect 17 out of the 36 standard metabolites over a time frame of 27 min, base peak electropherogram (BPE) is shown in **Fig. 2e**. All 16 amino acids were detected and separated from each other, with L-histidine and L-arginine being only detectable by CE (c.p. **Table 1**, HILIC ESI +/-). As shown in **Fig. 2e** (zoom), the isomeric amino acids L-leucine and L-isoleucine were baseline separated (20.1 and 20.4 min). Hence, this CE-MS method proved to be the most suitable method for the analysis of amino acids.

Typically anionic metabolites were analyzed in CE-MS using an alkaline BGE providing a strong electro-osmotic flow (EOF) transporting the analytes against the electrical field to the detector [3]. Both, NAD^+^ and NADH (nicotinamide adenine dinucleotide) were stable in the narrow pH range of 7.0–9.0 [4]. Thus, the pH of the alkaline BGE was adjusted to pH 8.5. Therefore, both analytes could be separated and analyzed using this method. The pH of 8.5 results in a relatively high EOF. This lead to the excellent separation of slow/medium migrating anions, whereas fast migrating anions were not detected. The BPE of the standard metabolites is shown in **Fig. 2b**. This method was capable to detect 30 out of 36 metabolites over a time frame of 30 min, 17 of them (such as neutral amino acids) co-migrating with the EOF (c.p. **Table 1**). Just basic amino acids were migrating as cations before the EOF and acidic amino acids were detected behind the EOF. Mainly, this is the method of choice when hexose-phosphates need to be separated (see **Fig. 2b, zoom**). All of the four standard hexose-phosphates were baseline separated, whereas all the other four (HILIC-MS and CE-MS) methods offered no (complete) separation (compare **Fig. 2a-e, zoom**).

To address the limitation of the alkaline BGE CE-MS method for fast migrating anions, a second CE-MS method for anion analysis was developed. The BGE pH of 2.1 suppressed the EOF, enabling the detection of anions at the anodic end of the capillary. However, only low pH-stable analytes and strong acids, which were (partly) dissociated at pH 2.1 could be analyzed. This method covered 15 of 36 analytes (4 co-migrating neutrals) over a time frame of 45 min (migration times are given in **Table 1**) and was capable to analyze phosphates and dicarboxylic acids. Two metabolites of the standard mix could be found exclusively using this method, namely tartrate and citrate.

# Tables

## S4: Yeast cell growth

**Table S4:** Results of the augmented multiplatform data analysis of yeast extracts based on suspects. Regulation calculated by the FC (indole treated/reference): FC < 0.50, down; 0.50 ≤ FC ≥ 2.00, stable; FC > 2.00, up. If a suspect appears only in reference cells, it is labeled as a knock-out. If a suspect appears only in treated cells, it is labeled as “induced”. The order of the presented results is the following: (i) augmentation ESI negative: HILIC (ESI-)/CE (alkaline, anion)/CE (acidic, anion), and (ii) augmentation ESI positive: HILIC (ES+)/CE (cation).

| **suspect** | **ESI negative augmentation** | | **ESI positive augmentation** | |
| --- | --- | --- | --- | --- |
|  | **[M-H]-** | **HILIC/CE (alkaline)/**  **CE (acidic)** | **[M+H]^+^** | **HILIC/CE (cation)** |
| Fumarate; Fumaric acid; trans-Butenedioic acid | 115.0031 | down / up / n.a. | 117.0188 | n.a. / n.a. |
| Oxaloacetate; Oxalacetic acid; Oxaloacetic acid; 2-Oxobutanedioic acid; 2-Oxosuccinic acid | 130.9980 | n.a. / n.a. / n.a. | 133.0137 | down / down |
| Aspartate/asparagine; L-Asparagine | 131.0457 | down / stable / n.a. | 133.0613 | down / down |
| (S)-Malate; L-Malate; L-Apple acid; L-Malic acid; L-2-Hydroxybutanedioic acid; Malate; Malic acid | 133.0137 | n.a. / n.a. / down | 135.0294 | n.a. / n.a. |
| 2-Oxoglutarate; Oxoglutaric acid; 2-Ketoglutaric acid; alpha-Ketoglutaric acid | 145.0137 | down / n.a. / n.a. | 147.0294 | down / up |
| L-Glutamine | 145.0613 | down / down / n.a. | 147.0770 | down / down |
| D-Xylose; L-Arabinose | 149.0450 | down / n.a. / knock out | 151.0607 | knock out / n.a. |
| Phosphoenolpyruvate; Phosphoenolpyruvic acid; PEP | 166.9745 | stable / n.a. / stable | 168.9902 | n.a. / induced |
| Glycerone-Phosphate; Glyceraldehyde-3-Phosphate; Glycerone phosphate; Dihydroxyacetone phosphate; 3-Hydroxy-2-oxopropyl phosphate; D-Glyceraldehyde 3-phosphate; (2R)-2-Hydroxy-3-(phosphonooxy)-propanal; Glyceraldehyde 3-phosphate | 168.9902 | n.a. / n.a. / n.a. | 171.0059 | n.a. / n.a. |
| N‐acetylleucine | 172.0974 | stable / n.a. / n.a. | 174.1131 | n.a. / n.a. |
| cis-Aconitate; cis-Aconitic acid | 173.0086 | up / n.a. / n.a. | 175.0243 | knock out / knock out |
| D-Glucosamine | 178.0716 | knock out / n.a. / n.a. | 180.0872 | down / induced |
| D-Sorbitol | 181.0713 | stable / stable / induced | 183.0869 | n.a. / n.a. |

**Table S4 continued:**

| **suspect** | **ESI negative augmentation** | | **ESI positive augmentation** | |
| --- | --- | --- | --- | --- |
|  | **[M-H]-** | **HILIC/CE (alkaline)/**  **CE (acidic)** | **[M-H]-** | **HILIC/CE (alk.)/**  **CE (aci.)** |
| 3-Phospho-D-glycerate; D-Glycerate 3-phosphate; 3-Phospho-(R)-glycerate; 3-Phosphoglycerate; 2-Phospho-D-glycerate; D-Glycerate 2-phosphate; 2-Phospho-(R)-glycerate - **Peak 1** | 184.9851 | knock out / n.a. / stable | 187.0008 | stable / n.a. |
| 3-Phospho-D-glycerate; D-Glycerate 3-phosphate; 3-Phospho-(R)-glycerate; 3-Phosphoglycerate; 2-Phospho-D-glycerate; D-Glycerate 2-phosphate; 2-Phospho-(R)-glycerate - **Peak 2** | 184.9851 | n.a. / n.a. / induced | 187.0008 | n.a. / n.a. |
| Oxalosuccinate; Oxalosuccinic acid | 189.0035 | n.a. / n.a. / n.a. | 191.0192 | down / induced |
| D-Glucuronate; D-Galacturonate | 193.0348 | n.a. / n.a. / n.a. | 195.0505 | n.a. / n.a. |
| N-Acetyl-D-mannosamine | 220.0822 | n.a. / n.a. / n.a. | 222.0978 | knock out / n.a. |
| Chitin; N-acetyl-D-glucosamine | 220.0822 | n.a. / n.a. / n.a. | 222.0978 | n.a. / n.a. |
| 2′‐Deoxycytidine | 226.0828 | stable / n.a. / n.a. | 228.0985 | n.a. / n.a. |
| beta-L-Arabinose 1-phosphate | 229.0113 | stable / n.a. / down | 231.0270 | n.a. / down |
| Thymidine | 241.0825 | stable / n.a. / n.a. | 243.0981 | induced / n.a. |
| L-Fucose-1-Phosphate | 243.0270 | down / n.a. / n.a. | 245.0427 | knock out / up |
| 2,4-Bis(acetamido)-2,4,6-trideoxy-beta-L-altropyranose; 2,4-Diacetamido-2,4,6-trideoxy-D-mannopyranose -**Peak 1** | 245.1138 | down / n.a. / n.a. | 247.1295 | stable / knock out |
| 2,4-Bis(acetamido)-2,4,6-trideoxy-beta-L-altropyranose; 2,4-Diacetamido-2,4,6-trideoxy-D-mannopyranose -**Peak 2** | 245.1138 | down / n.a. / n.a. | 247.1295 | knock out / n.a. |
| D-Glucosamine 6-phosphate; alpha-D-Glucosamine 1-phosphate - **Peak 1** | 258.0379 | stable / n.a. / induced | 260.0536 | stable / n.a. |
| D-Glucosamine 6-phosphate; alpha-D-Glucosamine 1-phosphate - **Peak 2** | 258.0379 | stable / n.a. / n.a. | 260.0536 | n.a. / n.a. |
| 3-Phospho-D-glyceroyl phosphate; 1,3-Bisphospho-D-glycerate; (R)-2-Hydroxy-3-(phosphonooxy)-1-monoan; 2,3-Bisphospho-D-glycerate; 2,3-Diphospho-D-glycerate; D-Greenwald ester; DPG | 264.9514 | stable / n.a. / n.a. | 266.9671 | knock out / n.a. |
| Arbutin; Ursin; Uvasol; Hydroquinone-O-beta-D-glucopyranoside | 271.0818 | knock out / stable / n.a. | 273.0975 | induced / n.a. |
| 1-Phospho-alpha-D-galacturonate; D-Glucuronate 1-phosphate | 273.0012 | knock out / n.a. / n.a. | 275.0168 | n.a. / n.a. |
| Salicin; Salicoside | 285.0975 | n.a. / n.a. / n.a. | 287.1131 | knock out / n.a. |
| N-Acetylmuramate | 292.1033 | n.a. / n.a. / n.a. | 294.1190 | n.a. / n.a. |

**Table S4 continued:**

| **suspect** | **ESI negative augmentation** | | **ESI positive augmentation** | |
| --- | --- | --- | --- | --- |
|  | **[M-H]-** | **HILIC/CE (alkaline)/**  **CE (acidic)** | **[M-H]-** | **HILIC/CE (alkaline)/**  **CE (acidic)** |
| N-Acetyl-D-mannosamine 6-phosphate | 300.0485 | down / down / down | 302.0641 | induced / n.a. |
| N-Acetyl-D-glucosamine 6-phosphate; N-Acetyl-alpha-D-glucosamine 1-phosphate | 300.0485 | n.a. / n.a. / n.a. | 302.0641 | n.a. / n.a. |
| N-Acetyl-Neuraminic Acid | 308.0982 | down / down / n.a. | 310.1139 | down / knock out |
| N-Glycoloyl-neuraminate | 324.0931 | n.a. / n.a. / n.a. | 326.1088 | n.a. / n.a. |
| Pseudaminic acid; N,N'-Diacetyllegionaminate | 333.1299 | n.a. / n.a. / n.a. | 335.1455 | n.a. / n.a. |
| beta-D-Fructose 1,6-bisphosphate | 338.9882 | n.a. / n.a. / induced | 341.0039 | n.a. / n.a. |
| Lactose, all disaccharide - **Peak 1** | 341.1084 | stable / stable / induced | 343.1241 | n.a. / n.a. |
| Lactose, all disaccharide - **Peak 2** | 341.1084 | induced / n.a. / n.a. | 343.1241 | n.a. / n.a. |
| Lactose, all disaccharide - **Peak 3** | 341.1084 | down / n.a. / n.a. | 343.1241 | n.a. / n.a. |
| AMP | 346.0553 | down / down / induced | 348.0709 | stable / n.a. |
| Arbutin 6-phosphate; Arbutin-6P | 351.0481 | n.a. / n.a. / n.a. | 353.0638 | n.a. / knock out |
| Salicin 6-phosphate; Salicin-6P | 365.0638 | knock out / n.a. / n.a. | 367.0795 | n.a. / n.a. |
| N-Acetylmuramic acid 6-phosphate ; N-Acetylmuramic acid alpha-1-phosphate | 372.0696 | knock out / n.a. / n.a. | 374.0853 | n.a. / knock out |
| N-Acetylneuraminate 9-phosphate | 388.0645 | n.a. / n.a. / n.a. | 390.0802 | knock out / n.a. |
| Chitobiose | 423.1616 | induced / n.a. / induced | 425.1772 | induced / n.a. |
| Thiamin diphosphate; Thiamine diphosphate; Thiamin pyrophosphate; TPP; ThPP | 424.0372 | stable / n.a. / n.a. | 426.0528 | up / n.a. |
| ADP | 426.0216 | down / n.a. / n.a. | 428.0373 | down / n.a. |
| Dolichyl phosphate | 439.2979 | n.a. / n.a. / n.a. | 441.3136 | n.a. / n.a. |
| 2-(alpha-Hydroxyethyl)thiamine diphosphate; 2-Hydroxyethyl-ThPP | 468.0634 | n.a. / n.a. / n.a. | 470.0791 | n.a. / n.a. |
| Dolichyl diphosphate | 519.2642 | n.a. / n.a. / n.a. | 521.2799 | n.a. / n.a. |
| 3-Carboxy-1-hydroxypropyl-ThPP; Succinate semialdehyde-thiamin diphosphate | 526.0689 | n.a. / n.a. / n.a. | 528.0846 | n.a. / n.a. |
| CDP-4-dehydro-3,6-dideoxy-D-glucose | 530.0577 | n.a. / n.a. / n.a. | 532.0734 | n.a. / n.a. |

**Table S4 continued:**

| **suspect** | **ESI negative augmentation** | | **ESI positive augmentation** | |
| --- | --- | --- | --- | --- |
|  | **[M-H]-** | **HILIC/CE (alkaline)/**  **CE (acidic)** | **[M-H]-** | **HILIC/CE (alkaline)/**  **CE (acidic)** |
| UDP-L-Ara4O | 533.0210 | n.a. / n.a. / n.a. | 535.0367 | n.a. / n.a. |
| UDP-L-Ara4N | 534.0526 | n.a. / n.a. / n.a. | 536.0683 | n.a. / n.a. |
| UDP-D-xylose; UDP-L-arabinose; UDP-apiose; UDP-L-arabinofuranose | 535.0367 | n.a. / n.a. / n.a. | 537.0523 | n.a. / n.a. |
| CDP-4-dehydro-6-deoxy-D-glucose | 546.0526 | n.a. / n.a. / n.a. | 548.0683 | n.a. / n.a. |
| UDP-4-dehydro-6-deoxy-D-glucose; UDP-4-keto-rhamnose | 547.0367 | n.a. / n.a. / n.a. | 549.0523 | n.a. / n.a. |
| UDP-L-rhamnose | 549.0523 | n.a. / n.a. / n.a. | 551.0680 | n.a. / n.a. |
| UDP-L-Ara4FN | 562.0476 | n.a. / n.a. / n.a. | 564.0632 | n.a. / n.a. |
| CDP-glucose | 564.0632 | n.a. / n.a. / n.a. | 566.0789 | n.a. / n.a. |
| UDP-glucuronate; UDP-D-galacturonate; UDP-L-iduronate | 579.0265 | n.a. / n.a. / n.a. | 581.0421 | n.a. / n.a. |
| GDP-4-dehydro-6-deoxy-D-mannose | 586.0588 | n.a. / n.a. / n.a. | 588.0744 | n.a. / n.a. |
| GDP-4-amino-4,6-dideoxy-alpha-D-mannose | 587.0904 | n.a. / n.a. / n.a. | 589.1061 | n.a. / n.a. |
| UDP-2-acetamido-4-dehydro-2,6-dideoxyglucose; UDP-2-acetamido-2,6-dideoxy-beta-L-arabino-hexos-4-ulose | 588.0632 | n.a. / n.a. / n.a. | 590.0789 | n.a. / n.a. |
| UDP-2-acetamido-4-amino-2,4,6-trideoxy-alpha-D-glucose;UDP-4-amino-4,6-dideoxy-N-acetyl-beta-L-altrosamine | 589.0949 | n.a. / n.a. / n.a. | 591.1105 | n.a. / n.a. |
| UDP-N-acetyl-alpha-D-glucosamine; UDP-N-acetyl-D-galactosamine; UDP-N-acetyl-D-mannosamine | 606.0738 | down / down / induced | 608.0894 | down / n.a. |
| UDP-2-acetamido-2-deoxy-alpha-D-ribo-hex-3-uluronate | 618.0374 | n.a. / n.a. / n.a. | 620.0530 | n.a. / n.a. |
| GDP-D-mannuronate | 618.0486 | n.a. / n.a. / n.a. | 620.0643 | n.a. / n.a. |
| UDP-2-acetamido-3-amino-2,3-dideoxy-alpha-D-glucuronate | 619.0690 | n.a. / n.a. / n.a. | 621.0847 | n.a. / n.a. |
| UDP-N-acetyl-2-amino-2-deoxy-D-glucuronate; UDP-N-acetyl-D-mannosaminouronate; UDP-N-acetyl-D-galactosaminuronic acid | 620.0530 | n.a. / n.a. / n.a. | 622.0687 | n.a. / n.a. |
| UDP-6-sulfoquinovose | 629.0091 | n.a. / n.a. / n.a. | 631.0248 | n.a. / n.a. |
| GDP-4-acetamido-4,6-dideoxy-alpha-D-mannose | 629.1010 | n.a. / n.a. / n.a. | 631.1167 | n.a. / n.a. |
| CMP-N-glycoloylneuraminate | 629.1344 | n.a. / n.a. / n.a. | 631.1501 | n.a. / n.a. |
| UDP-2,4-bis(acetamido)-2,4,6-trideoxy-beta-L-altropyranose; UDP-N,N'-diacetylbacillosamine | 631.1054 | n.a. / n.a. / n.a. | 633.1211 | n.a. / n.a. |

**Table S4 continued:**

| **suspect** | **ESI negative augmentation** | | **ESI positive augmentation** | |
| --- | --- | --- | --- | --- |
|  | **[M-H]-** | **HILIC/CE (alkaline)/**  **CE (acidic)** | **[M-H]-** | **HILIC/CE (alkaline)/**  **CE (acidic)** |
| CMP-pseudaminic acid; CMP-N,N'-diacetyllegionaminate | 638.1712 | n.a. / n.a. / n.a. | 640.1868 | n.a. / n.a. |
| UDP-2,3-diacetamido-2,3-dideoxy-alpha-D-glucuronate; UDP-2,3-diacetamido-2,3-dideoxy-alpha-D-mannuronate | 661.0796 | induced / n.a. / n.a. | 663.0953 | n.a. / n.a. |
| UDP-N-acetyl-3-(1-carboxyvinyl)-D-glucosamine | 676.0793 | n.a. / n.a. / n.a. | 678.0949 | n.a. / n.a. |
| UDP-N-acetylmuramate | 678.0949 | n.a. / n.a. / n.a. | 680.1106 | n.a. / n.a. |
| Acetyl-CoA; Acetyl coenzyme A | 808.1180 | n.a. / n.a. / n.a. | 810.1337 | stable / n.a. |
| Succinyl-CoA; Succinyl coenzyme A | 866.1235 | n.a. / n.a. / n.a. | 868.1392 | n.a. / n.a. |
| Undecaprenyl phosphate alpha-L-Ara4N | 976.7164 | n.a. / n.a. / n.a. | 978.7320 | n.a. / n.a. |
| **number of detected targets** |  | 38 |  | 31 |

# Figures

## S1: Yeast cell growth

**Fig. S1:** Yast cell growth of Mock and Effect 1

## S2: *AriumMS* GUI

**A**

**B**


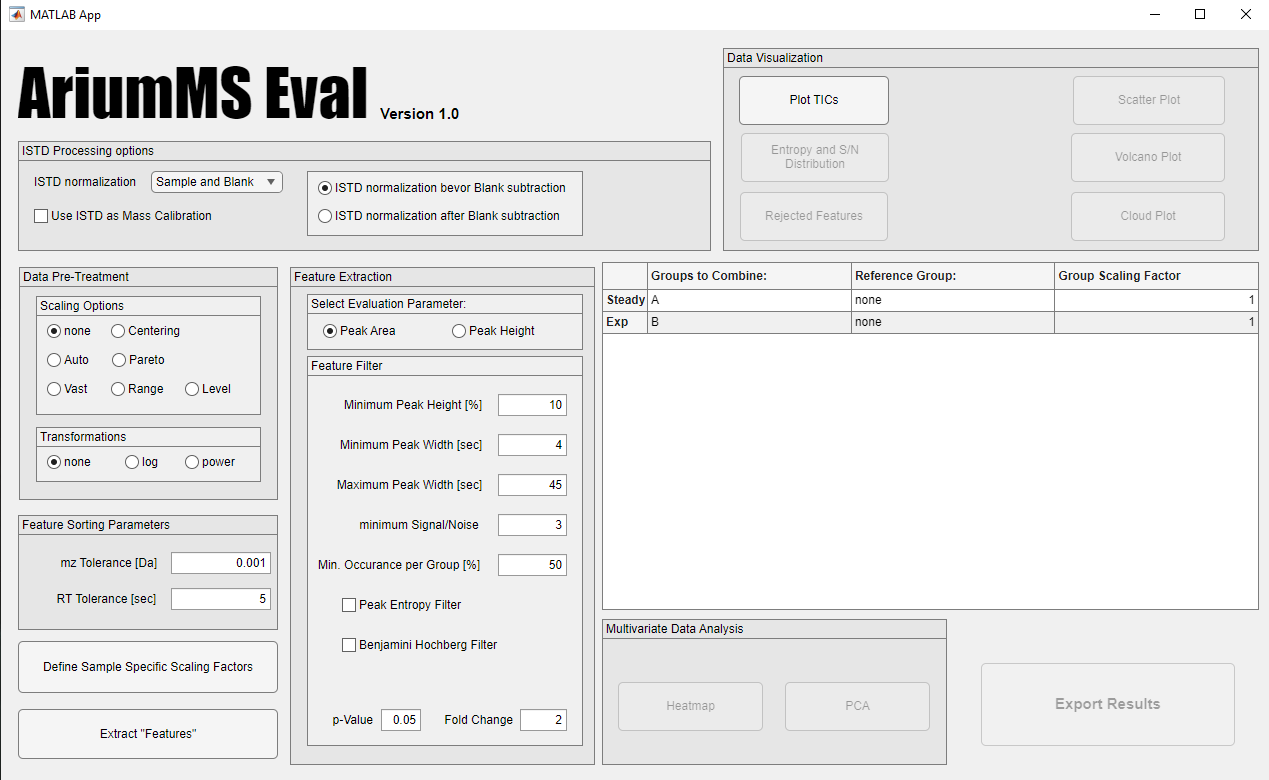

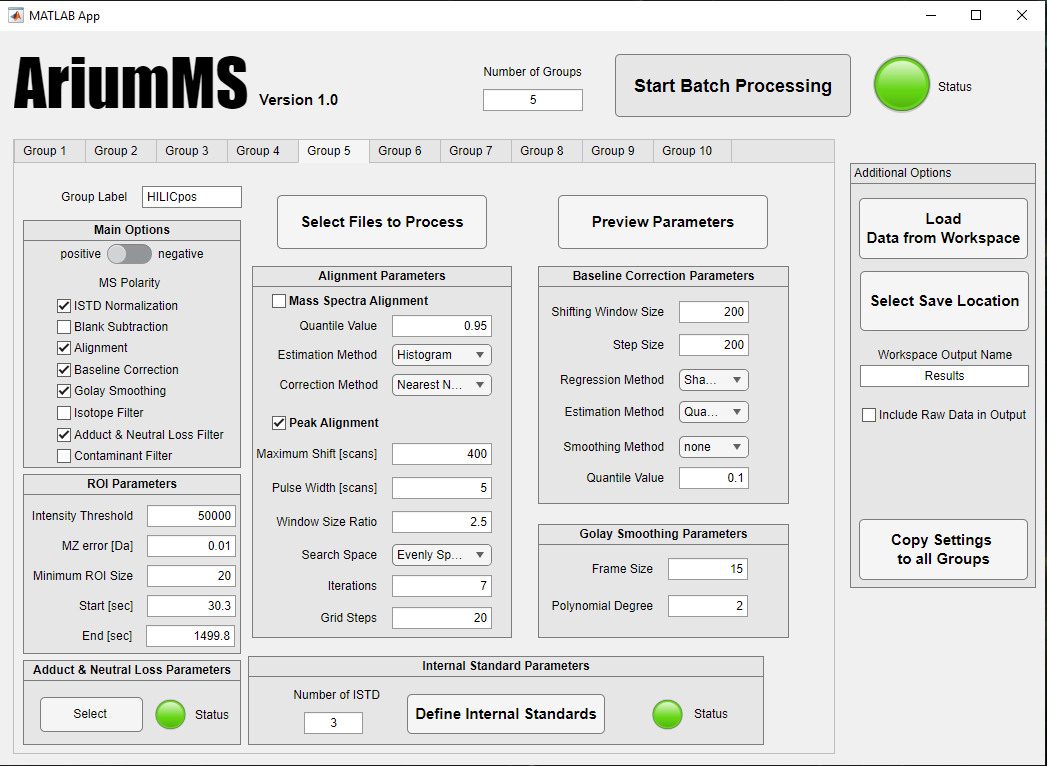


**Fig. S2:** Graphical user interface of *AriumMS* (A) and *AriumMSEval* (B)

## S3: *AriumMS* processing workflow

**
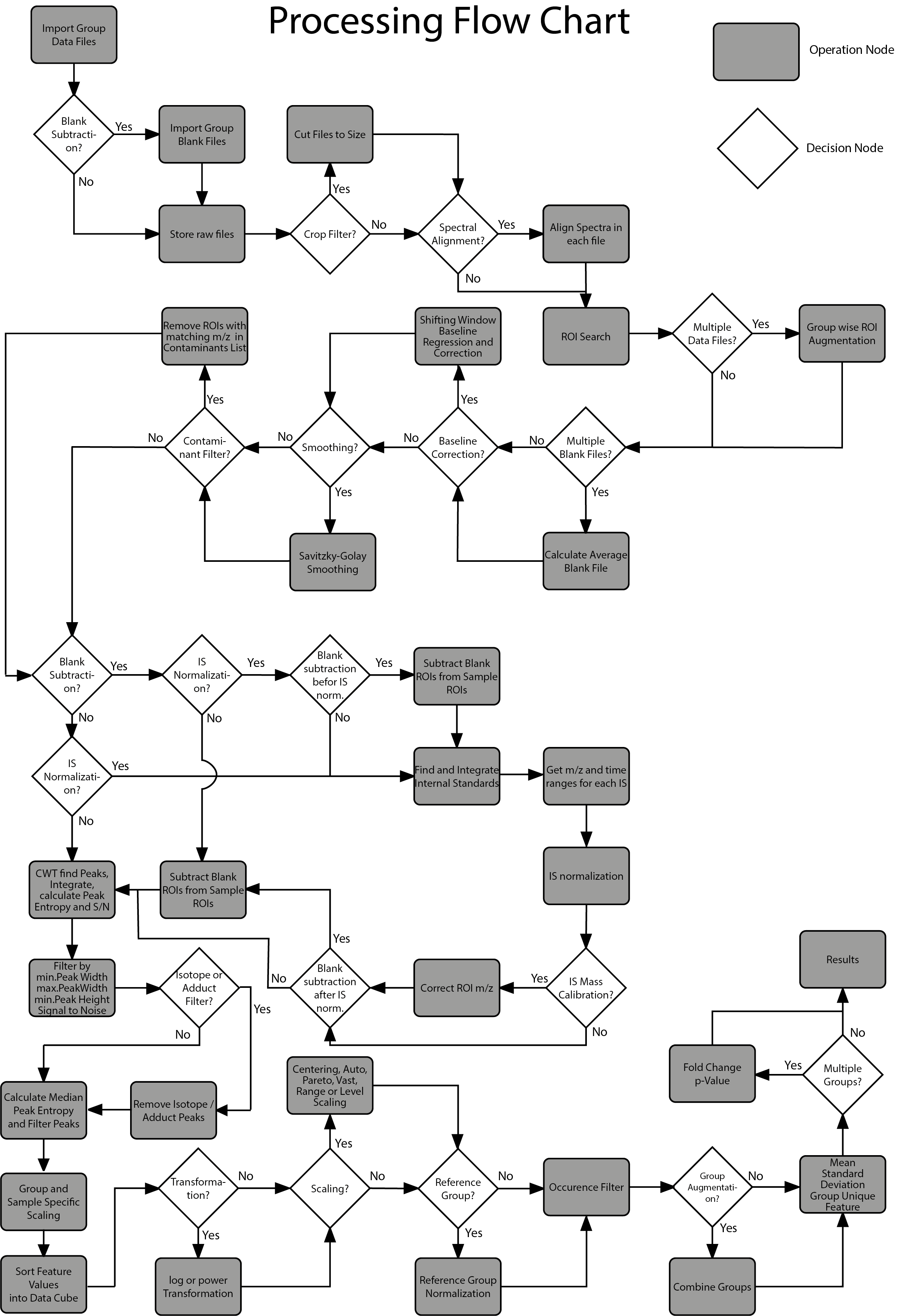
**

**Fig. S3:** Detailed flowchart of *AriumMS*

## S4: Yeast metabolomics, observed effects

**
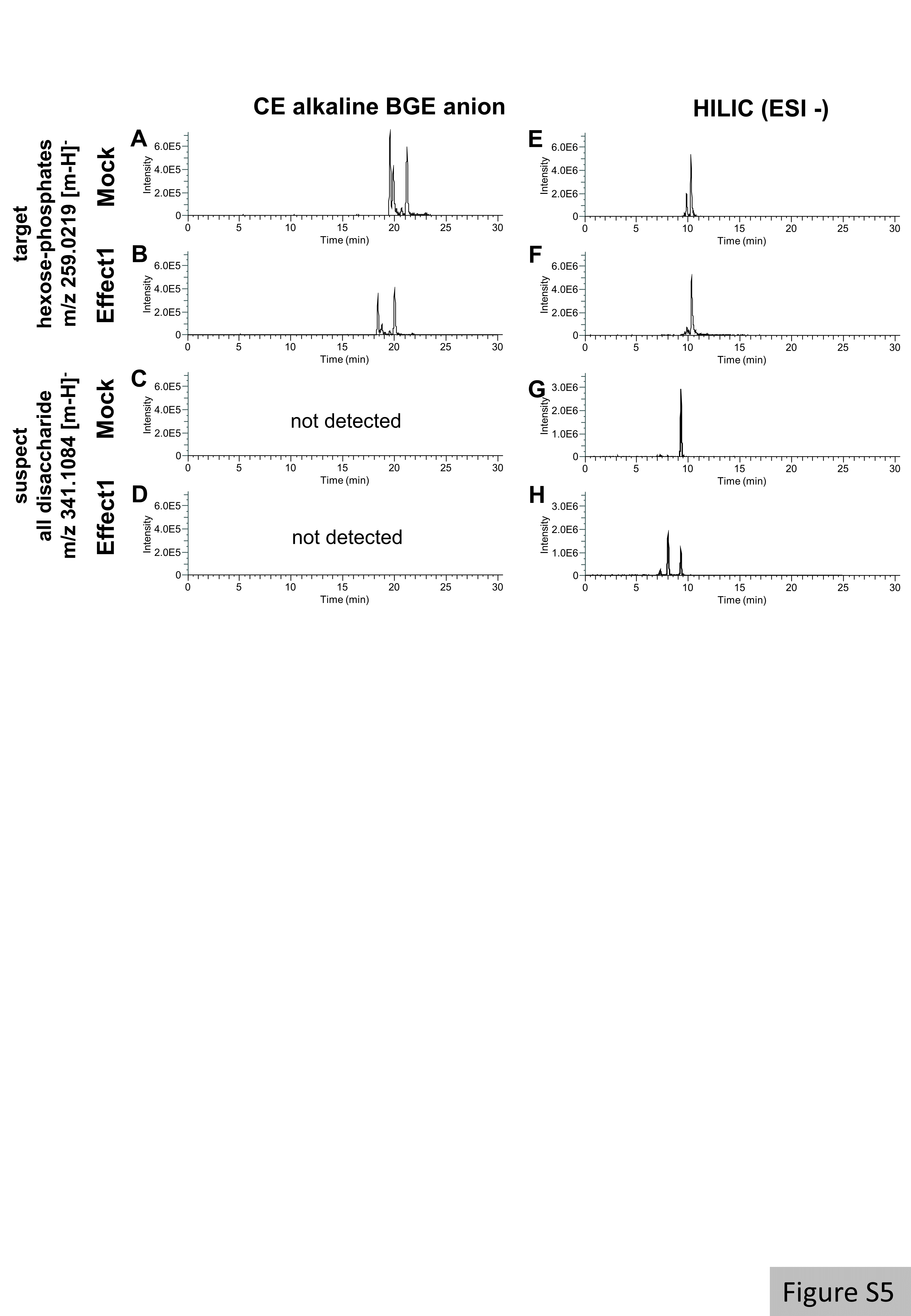
**

**Fig. S4:** Observed effects on the change in sugar metabolism derived from the treatment of the yeast cells. (A-D) shows the EIEs of the CE alkaline anion method and (E-F) the EICs of the HILIC anion method. EIC/Es of the hexose-phosphates are given in (A-B+E-F) and of the disaccharides in (C-D+G-H).

## S5: PCA of yeast extracts


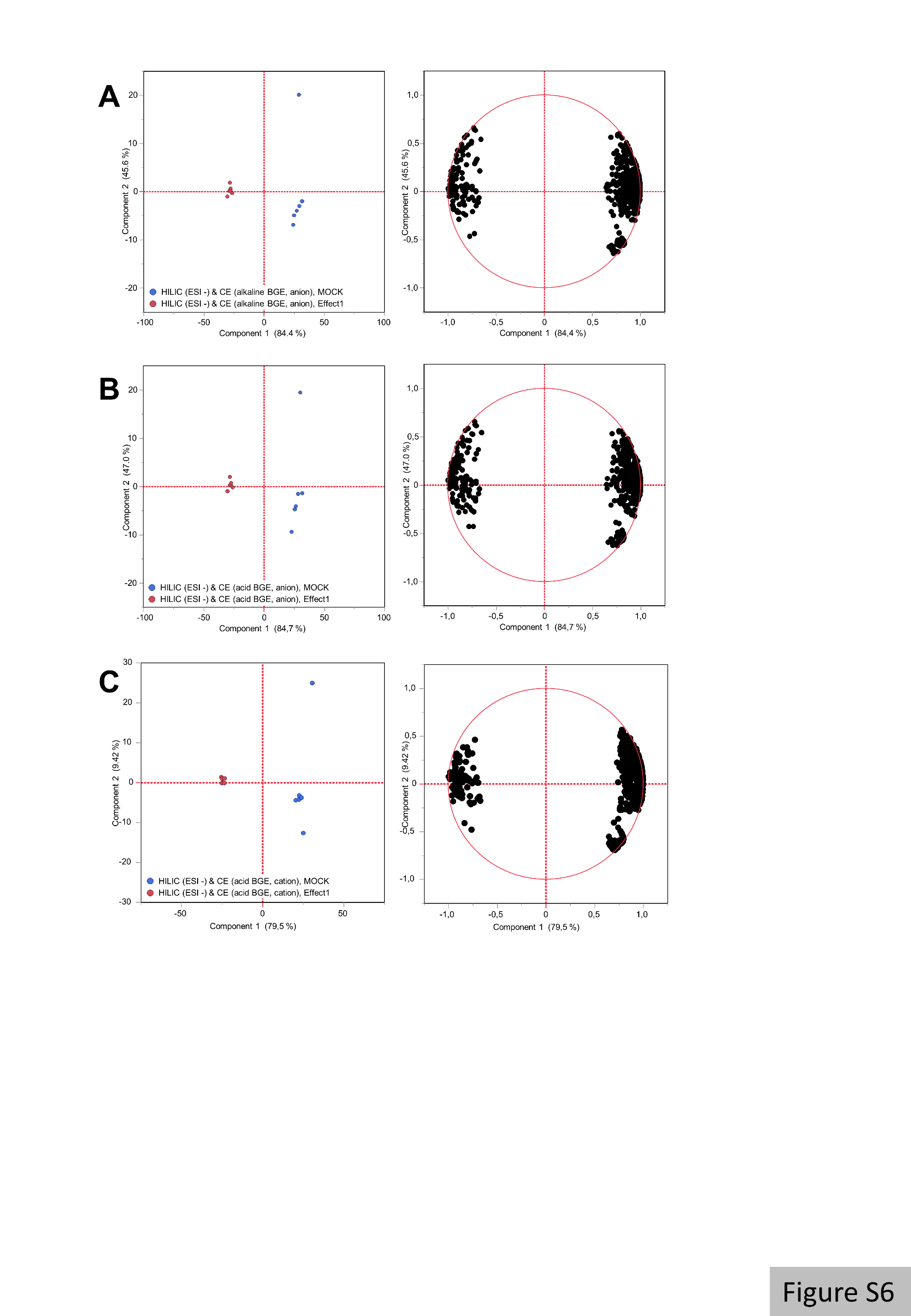


**Fig. S5:** PCA of the three augmentations containing remaining features after Benjamini Hochberg filter. (A) shows the PCA of HILIC (ESI-) & CE (alkaline BGE, anion), (B) of HILIC (ESI-) & CE (acidic BGE, anion), and (C) of HILIC (ESI+) & CE (cation).

# References

1. Chambers, M. C.; Maclean, B.; Burke, R.; Amodei, D.; Ruderman, D. L.; Neumann, S.; Gatto, L.; Fischer, B.; Pratt, B.; Egertson, J.; Hoff, K.; Kessner, D.; Tasman, N.; Shulman, N.; Frewen, B.; Baker, T. A.; Brusniak, M.-Y.; Paulse, C.; Creasy, D.; Flashner, L.; Kani, K.; Moulding, C.; Seymour, S. L.; Nuwaysir, L. M.; Lefebvre, B.; Kuhlmann, F.; Roark, J.; Rainer, P.; Detlev, S.; Hemenway, T.; Huhmer, A.; Langridge, J.; Connolly, B.; Chadick, T.; Holly, K.; Eckels, J.; Deutsch, E. W.; Moritz, R. L.; Katz, J. E.; Agus, D. B.; MacCoss, M.; Tabb, D. L.; Mallick, P. A cross-platform toolkit for mass spectrometry and proteomics. Nat. Biotechnol. 2012, 30 (10), 918–920. DOI: 10.1038/nbt.2377.
2. Pluskal, T.; Castillo, S.; Villar-Briones, A.; Oresic, M. MZmine 2: modular framework for processing, visualizing, and analyzing mass spectrometry-based molecular profile data. BMC Bioinf. 2010, 11 (395), 1–11. DOI: 10.1186/1471-2105-11-395.
3. Sawada, H.; Nogami, C. Capillary electrophoresis–electrospray ionization mass spectrometry using uncoated fused-silica capillaries and alkaline buffer solution for the analysis of small carboxylic acids. Anal. Chim. Acta 2004, 507 (2), 191–198. DOI: 10.1016/j.aca.2003.11.047.
4. Sato, S.; Yanagisawa, S. Capillary electrophoresis–electrospray ionization-mass spectrometry using fused-silica capillaries to profile anionic metabolites. Metabolomics 2010, 6 (4), 529–540. DOI: 10.1007/s11306-010-0223-x.
